# Service-oriented vulnerability assessment for the larger-scale high speed railway infrastructure network: a case in China

**DOI:** 10.1038/s41598-026-43851-8

**Published:** 2026-03-19

**Authors:** Hanqing Zhang, Hongfei Xing, Xiaoping Ma, Limin Jia

**Affiliations:** 1 School of Traffic and Transportation, Beijing Jiaotong University, Beijing, 100044 China; 2 State Key Laboratory of Advanced Rail Autonomous Operation, Beijing, 100044 China; 3 Beijing Aerospace Xinli Technology Co., Ltd, Beijing, 100000 China

**Keywords:** High speed railway network, Vulnerability assessment, Passenger demand estimation, Passenger flow transfer, Engineering, Mathematics and computing

## Abstract

High-speed rail (HSR) vulnerability refers to the sensitivity of the infrastructure network to performance degradation under disruptions. Due to the increasing complexity of modern railway systems, topological analysis alone is no longer sufficient to capture their actual operational behavior. Recent studies have increasingly recognized the importance of integrating network functionality with topological structures when assessing vulnerability. This paper proposes a service-oriented vulnerability assessment framework that evaluates HSR vulnerability from the perspective of passenger travel service capacity under uncertain interruptions. A three-layer coupled network is constructed based on a bipartite graph, mapping passenger demand onto infrastructure via a functional layer to capture the coupling between passengers, trains, and tracks. The proposed framework integrates service-based vulnerability metrics, a novel passenger demand evaluation method, and realistic transfer strategies. A case study of China’s HSR network demonstrates the method’s effectiveness. Results show that the network generally exhibits low vulnerability due to strong connectivity. However, lines serving cities with high passenger volumes or involving long-distance transfers are more vulnerable under full-load conditions, where limited spare capacity may force passengers to choose alternative transport modes. These results provide meaningful insights for enhancing disaster prevention and emergency management in high-speed railway systems, emphasizing that the degradation of service capacity should also be regarded as a critical dimension when assessing structural vulnerability.

## Introduction

High-speed railways are playing an increasingly important role in passenger transportation nowadays in several countries. In China, according to the National Railway Administration’s disclosure in November 2025, by the end of 2024, the total operating mileage of railways nationwide reached 162,000 km, with high-speed railways accounting for 48,000 km, representing approximately 29.6% of the total operating mileage^1^. In 2024, the total number of railway passengers nationwide reached 4.312 billion, of which 3.272 billion were on bullet trains (mainly high-speed rail-related models). Bullet train passenger volume accounted for 75.9% of the total railway passenger volume that year, highlighting the core position of high-speed rail in railway passenger transport^2^.

Under the primary driving force of global warming, compounded by excessive resource exploitation and unregulated urban expansion in recent years, the impact of natural disasters on China’s railway network has gradually shifted from low-frequency to high-frequency events, with increasing levels of destruction. For example, snowstorms in the winter of 2008 destroyed a key railway line between Beijing and Guangzhou while half a million passengers gathered in front of Guangzhou Railway station, causing a serious stampede^3^.

Therefore, a key aspect of railway system safety assessment is the accurate analysis of the changes in operational efficiency caused by disruptions or failures due to disruptions. To date, multiple studies have been conducted on the capability of HSR with the main topic of vulnerability, robustness and resilience^4–9^. These studies primarily adopted the theory of complex network to identify the key stations in railway infrastructure network and evaluate the service capability of the topological HSR under different degrees of attacks. Although network-structure-based analytical approaches can rapidly identify critical stations or links within the network, empirical observations in real-world high speed railway operations reveal that the failure of certain structurally “central” nodes does not necessarily lead to severe system-wide consequences. This discrepancy arises because such purely graph-theoretical methods overlook the fundamental functions and core mission of high speed railway systems-namely, to ensure safe, efficient, and reliable passenger transportation. Consequently, the extent to which passenger travel demands are fulfilled constitutes a central metric for evaluating the operational efficiency of high-speed rail systems. Therefore, analyses that rely solely on network topology exhibit inherent limitations. To address this limitation, some scholars tried to identify the key stations in the HSR from the perspective of passenger services, considering the trains schedule and the demand of passengers^10,11^.

However, in these studies, the passengers’ demands were represented by the number of the trains which stops at the stations. Besides, the existing studies mostly estimated the passenger flow using the train schedules, while in which just the stopover trains are included and without incorporating the trains which pass but did not stop at the stations. This will underestimate the passenger flow and reduce the accuracy of evaluation model. Moreover, under real-world operational conditions, railway lines are more susceptible to extreme weather events due to their long spatial extent and limited shielding, making them inherently more vulnerable than stations, which typically involve higher construction costs. Therefore, conducting vulnerability analysis of railway infrastructure networks from the perspective of passenger service offers greater academic significance and practical value. Based on this, we propose service-oriented vulnerability.

Building upon this perspective, this study proposes an integrated, service-oriented framework for large-scale vulnerability assessment of high-speed railway systems under disruption scenarios. The proposed framework is distinguished not only by enhanced realism, but more importantly by its systematic integration of infrastructure, train operations, and passenger demand, as well as its scalability and decision relevance at the national level.

One key contribution of this study lies in the explicit coupling of physical infrastructure, train services, and passenger demand through a three-layer network model. By introducing a function network derived from nationwide timetables as an intermediate layer, the framework establishes a direct mapping between railway line segments and actual train operations. This design allows vulnerability to be evaluated in terms of service performance and passenger flow impacts, rather than relying solely on abstract connectivity measures that dominate much of the existing literature.

A second contribution is the development of a Passenger Demand Estimation Method based on nationwide timetable information. Under realistic data constraints, this method provides a consistent representation of passenger demand by jointly considering stopping patterns and service capacity. Compared with conventional schedule-based or topology-driven demand approximations, the proposed method improves coherence between demand estimation and operational planning principles, thereby enhancing the interpretability and credibility of vulnerability assessment results.

Third, this study introduces a Passenger Transfer Strategy to explicitly model passenger accommodation under disruption scenarios. Instead of assuming complete demand loss following infrastructure failures, the proposed strategy evaluates whether disrupted passengers can still complete their intended journeys through feasible direct or one-transfer alternatives within the remaining network. This enables the framework to distinguish between unrecoverable passenger loss and recoverable service capacity, extending existing vulnerability models by incorporating service adaptability and demand redistribution potential.

From an application perspective, the proposed framework offers strong practical value. It relies exclusively on publicly obtainable infrastructure and timetable data and can therefore be readily applied at the national scale without requiring individual-level passenger trajectories or behavioral parameters. Moreover, the resulting vulnerability indicators, expressed in terms of passenger service loss and recoverable demand, provide intuitive and decision-oriented information that directly supports infrastructure prioritization, emergency preparedness, and resilience planning. Through these features, the proposed framework systematically extends existing research by providing a comprehensive, scalable, and operationally meaningful approach to railway infrastructure vulnerability assessment.

The rest of this paper is organized as follows: section “Literature review” reviews the related literatures; section “Methodology” presents a passenger services-oriented HSR vulnerability evaluation model. The case study is conducted in section “Case study” and “Result and discussion” to analyze the vulnerability of China’s HSR from the perspective of network structure and evaluation indicators. The conclusions, implications and limitations are illustrated in section “Conclusion and suggestions”.

## Literature review

The complexity of the high-speed railway network is not only reflected in the increasingly complex structure of the infrastructure network, but also reflected in the operation of the traffic flow and the distribution of passenger flow. With the improvement of infrastructure network, the study of network topology^12–14^ cannot improve the efficiency of the network, especially when the network is facing sudden disasters. Therefore, it is necessary to further analyze the high-speed railway network based on the train schedule and the travel demand of passengers, to provide reasonable suggestions for the formulation of emergency plans and the allocation of disaster relief resources.

Although numerous studies have investigated road transportation networks^15–18^ and aviation networks^19–21^ from the perspectives of travel demand or network topology, these approaches cannot be directly applied to railway network analysis.

Compared with road transportation networks, railway operations are strictly constrained by fixed timetables, whereas road vehicles generally possess much greater flexibility in route choice. In contrast to aviation networks, trains must pass through all intermediate stations along a railway corridor between their origin and terminal stations, while aircraft typically travel directly between origin and destination airports without intermediate stops. These fundamental operational differences imply that vulnerability modeling approaches developed for road or aviation networks are not fully suitable for railway networks and require dedicated methodological adaptations.

Due to the reason of data confidentiality, the research on the vulnerability of HSR based on passenger flow demand usually needs to estimate passenger flow from the schedule. P-space network^22^ is widely used to estimate the demand based on train schedule. In p-space network, the nodes represent the stations, and two stations will be connected if a train stops at the two stations. The weight indicates the amounts of trains between the two stations, which is usually used to represent the demand. Apparently, a train stops at n stations can generate lines, and it will lead a huge gap between trains with fewer stops and trains with more stops. There is 45 times more demand for a train with 10 stops than a train with 2 stops, which is not reasonable in the real operation of trains.

Most studies on HSR can only evaluate the vulnerability of the stations, either from the aspect of infrastructure network or passenger flow demand. Scholars^11,23^ used complex network theory to evaluate the structural vulnerability of high-speed railway network when stations were attacked. Scholars^24^ analyzed the robustness of China’s high-speed rail when stations and trains were attacked from the perspective of demand. However, these approaches mainly focus on structural or station-level responses and therefore cannot fully capture how infrastructure disruptions affect passenger flows or how service degradation propagates across the network. As a result, they fall short in evaluating vulnerability from the perspective of passenger service capacity, which is essential for understanding the real operational impact of disruptions.

Recent research has increasingly emphasized passenger-centered and system-based perspectives in railway vulnerability assessment, recognizing that network criticality cannot be explained by topology alone. Scholars^25^ developed a Railway Network Vulnerability Model (RNVM) that jointly considers infrastructure availability, train operations, and dynamic passenger routing under disruptions, demonstrating that critical links are highly demand-dependent and shaped by service-level adjustments. Similarly, passenger-oriented disruption management studies^26^ show that vulnerability is influenced by multi-criteria travel behavior—including travel time, transfer burden, comfort, and operational interventions—and that passengers may reroute or lose connectivity even when the physical network remains structurally intact. These studies collectively highlight that railway vulnerability is a multi-layer, service-driven process arising from interactions among infrastructure capacity, operational decisions, and passenger demand.

Beyond the railway domain, related research in urban rail transit systems has also demonstrated the importance of integrating service performance into vulnerability assessment and reduction strategies. In particular, the study^27^ showed that a network’s vulnerability can be effectively mitigated by optimally allocating protective resources to stations and links while accounting for disruption extent and severity. Their scenario-based evaluation revealed that weighted travel time and passenger losses can be significantly reduced through targeted protection strategies. These findings reinforce the view that vulnerability should be understood not only through structural degradation but also through its impact on passenger mobility and service continuity, further supporting the service-oriented perspective adopted in the present study.

Based on these insights, this study focuses on how infrastructure disruptions affect passenger flow and assesses network vulnerability from a service-oriented perspective. Our approach integrates train service networks and infrastructure design networks into a graph-structured framework using a multi-layered coupled network, thus combining structural analysis with demand- and service-oriented characteristics. This enables us to identify infrastructure network disruption events that significantly reduce passenger capacity.

Therefore, compared with existing vulnerability assessment approaches, the proposed framework preserves essential features of railway service interactions while enabling a more suitable and rapid evaluation. In particular, railway services are represented as timetable-constrained operations rather than abstract flows, and passenger service performance is evaluated in terms of service capacity degradation instead of train counts alone. Passenger demand is generated using a timetable-driven estimation method that accounts for both stopping patterns and service capacity, which avoids the systematic underestimation of passenger flows commonly found in schedule-based approximations that consider only stopping trains.

Moreover, service interactions are explicitly linked to the availability of individual railway line segments, allowing infrastructure disruptions to be evaluated through their direct impacts on passenger services. Under disruption scenarios, passenger accommodation is not treated as complete demand loss but is instead captured through feasible reassignment via alternative services. Importantly, the computational efficiency of the framework is improved through optimization within the Passenger Transfer Strategy, where matrix-based representations are employed to replace exhaustive sequential searches when identifying feasible transfer options. This design substantially reduces computational complexity and enables rapid vulnerability screening at the national scale, while retaining key operational and demand-related characteristics of real-world railway systems.

As a result, this study constructs a three-layer network framework consisting of a physical network, a function network, and a demand network to support vulnerability analysis of the high-speed railway system. The physical network represents the railway infrastructure topology and describes the connectivity of railway line segments. The demand network is used to estimate passenger volumes associated with train services, while accounting for differences in stopping patterns and service capacity. The function network serves as an intermediate layer that maps timetable-based train services onto the physical infrastructure, enabling passenger demand to be allocated to specific railway line segments.

Based on this framework, a passenger service–oriented vulnerability assessment is conducted. The performance of the high-speed railway system is evaluated in terms of the number of passengers who can successfully complete their trips, and vulnerability is quantified by comparing system performance before and after line disruption scenarios. Passenger transfer mechanisms are incorporated to represent passenger accommodation under disruptions. Using this approach, the vulnerability of individual railway line segments in China’s high-speed railway network is evaluated and visualized, providing a system-level overview of infrastructure sensitivity to service disruptions.

## Methodology

This study proposes a vulnerability assessment method for HSR infrastructure network from a passenger service-oriented perspective. Compared with conventional approaches based on complex network theory, the proposed method provides a more intuitive representation of the actual operational state of the railway system when certain regions are disrupted.

**Fig. 1:**
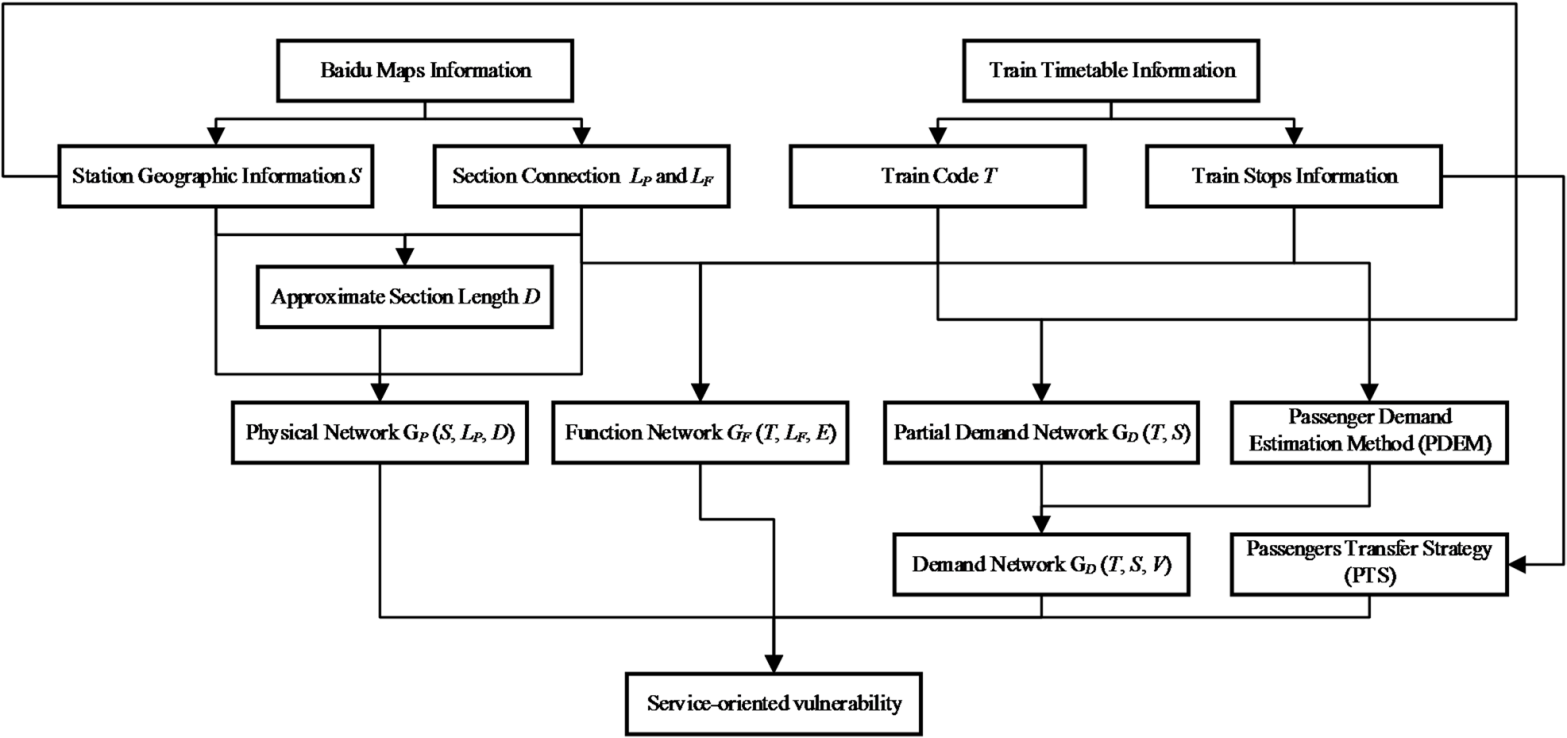
Framework of the service-oriented vulnerability assessment for HSR infrastructure network.

To evaluate the service-oriented vulnerability of the high-speed railway network, this study develops a methodological framework that integrates multi-source data, a three-layer coupled network model, and two key analytical components: the Passenger Demand Estimation Method (PDEM) and the Passenger Transfer Strategy (PTS). As illustrated in the framework diagram below, the overall process begins with the extraction of station geographic information, section connectivity, train timetable information, and train stopping patterns. These data are subsequently used to construct the Physical Network, the Function Network, and the Demand Network, which together capture the structural layout, service operations, and passenger demand characteristics of the railway system. PDEM maps timetable-derived service frequency information onto the demand layer, while PTS evaluates the extent to which disrupted passengers can be reassigned through alternative routes following a line failure. The integration of these components enables a comprehensive assessment of how infrastructure disruptions propagate through service and demand layers, ultimately affecting the passenger-carrying capability of the national high-speed railway system. The overall methodological structure is summarized in the framework diagram shown below (Fig. 1).

Accordingly, the first step involves constructing a three-layer HSR network model to reveal the hierarchical logical relationship among structure, function, and service from a system-level perspective. This enables the coupling and mapping from passenger demand to physical infrastructure and provides multi-dimensional indicator support for vulnerability assessment and recovery strategies (section “Three-layer network model of HSR”). Subsequently, based on the constructed three-layer coupled HSR network, a passenger service-oriented vulnerability assessment framework is developed. By accurately estimating passenger travel demand and transfer willingness, this model enables the evaluation of HSR system performance under disruption from the perspective of satisfying passenger needs (section “Passenger service-oriented HSR vulnerability assessment framework”).

The key symbols and parameters used in the three-layer network model and subsequent analytical procedures are summarized in Table 1.

**Table 1:** The parameter table.

Symbol	Meaning	Layer/Section
G_*P*_ (*S*, *L*_*P*_, *D*)	Physical Network	Three-layer Network
*G*_*F*_ (*T*, *L*_*F*_, *E*)	Function Network	Three-layer Network
G_*D*_ (*T*, *S*, *V*)	Demand Network	Three-layer Network
*S*	Node set of stations	Physical & Function Network
*L*_*P*_	Edge set of railway lines	Physical Network
*D*	Distance between two stations	Physical Network
*T*	Node set of trains	Function & Demand Network
*L*_*F*_	Node set of railway lines	Function Network
*E*	Edge set representing the connection between trains and lines	Function Network
*V*	Edge set representing the number of passengers boarding a train at a station	Demand Network
*C*	capacity of a train	PDEM
*p*	proportion of passengers at the departure station	PDEM
*V*_*max*_	a maximum passenger load per train at a station	PDEM
*V*_*min*_	a minimum passenger load per train at a station	PDEM
${T_S}$	set of all trains stop at station *S*	PDEM
$\left| {{T_S}} \right|$	the number of trains stop at stations *S*	PDEM
$H\left( {{T_S}} \right)$	the set of trains departing from station *S*	PDEM
$T\left( {{T_S}} \right)$	the set of trains terminating at station *S*	PDEM
$I\left( {{T_S}} \right)$	the set of trains stopping at station *S* as an intermediate station	PDEM
${V_S}$	The number of passengers at station *S*	PDEM
${V_t}$	he number of passengers of train *t*	PDEM
${S_t}$	stops in the schedule of train *t*	PDEM & Passengers Transfer Strategy
m	number of track segments in HSR infrastructure network	Passengers Transfer Strategy
n	number of trains passing through a line	Passengers Transfer Strategy
P	number of trains stopping at a given station	Passengers Transfer Strategy
q	number of stations served by each train	Passengers Transfer Strategy
$O\left( * \right)$	Computational complexity	Passengers Transfer Strategy
$n^{\prime}$	the number of stations	Passengers Transfer Strategy
$\alpha$	the number of trains in the timetable	Passengers Transfer Strategy
$\beta$	the number of stops of the trains in the timetable	Passengers Transfer Strategy
${\boldsymbol{M}}~\left( {{{\boldsymbol{s}}_{\boldsymbol{i}}},~{{\boldsymbol{s}}_{\boldsymbol{j}}}} \right)$	city reachability matrix	Passengers Transfer Strategy
$V_{t}^{{transfer}}$	The number of passengers can be converted from train t	Passengers Transfer Strategy
${T_l}$	the affected train set	Passengers Transfer Strategy
${O_t}$	The originating stations of the affected trains	Passengers Transfer Strategy
${D_t}$	The terminal stations of the affected trains	Passengers Transfer Strategy
$T\left( {{O_t}} \right)$	the set of trains stopping at station ${O_t}$	Passengers Transfer Strategy
${t_{{O_t}}}$	Trains in set $T\left( {{O_t}} \right)$	Passengers Transfer Strategy
$S\left( {{t_{{O_t}}}} \right)$	the set of stations not yet served by train ${t_{{O_t}}}$	Passengers Transfer Strategy
${s^{transfer}}$	the potential transfer station	Passengers Transfer Strategy
$RV$	the vulnerability of HSR	Vulnerability Assessment
${P^{loss}}$	the loss of passengers caused by the failure of lines	Vulnerability Assessment
${P^{normal}}$	the number of passengers in normal operation	Vulnerability Assessment
${P^{affected}}$	the number of affected passengers caused by disruption	Vulnerability Assessment
${P^{transfer}}$	the number of passengers can be transferred	Vulnerability Assessment
$\theta$	a generic model parameter	Sensitivity analysis
${S_\theta }$	parameter perturbation stability	Sensitivity analysis
${\rho _\theta }$	Identification and ranking changes of critical paths under parameter perturbations	Sensitivity analysis

### Three-layer network model of HSR

To thoroughly investigate the vulnerability of HSR infrastructure networks under *unexpected disruptions*, it is essential to first construct an integrated network model that can faithfully represent the system’s actual operational state. This forms the foundation and key prerequisite for conducting a systematic vulnerability assessment.

This study introduces an innovative three-layer coupled network model, composed of a Physical Network, Function Network, and Demand Network, to comprehensively capture the characteristics of the HSR system in terms of structural layout, transport capacity, and responsiveness to passenger demand.

1. The Physical Network focuses on the topological relationships among railway lines, stations, and other infrastructure elements, representing the static configuration of the HSR system;
2. The Function Network models the train operation plans and actual transport capacities, serving as the dynamic functional projection of the physical structure;
3. The Demand Network reflects the intensity of service and transport pressure borne by different stations and segments during actual operations, based on passenger travel demand;

The logical mapping and functional dependencies among these three networks are realized through a bipartite graph modeling approach, which enables the integrated system to capture multidimensional response characteristics of the HSR under uncertain disruptions (e.g., natural disasters, equipment failures). Compared to traditional methods that rely solely on structural topology, the proposed model simultaneously considers structural rationality, transport functionality, and service adaptability. It thus provides a robust theoretical and data-driven foundation for advanced dynamic vulnerability assessments and the development of effective recovery strategies.

**Fig. 2:**
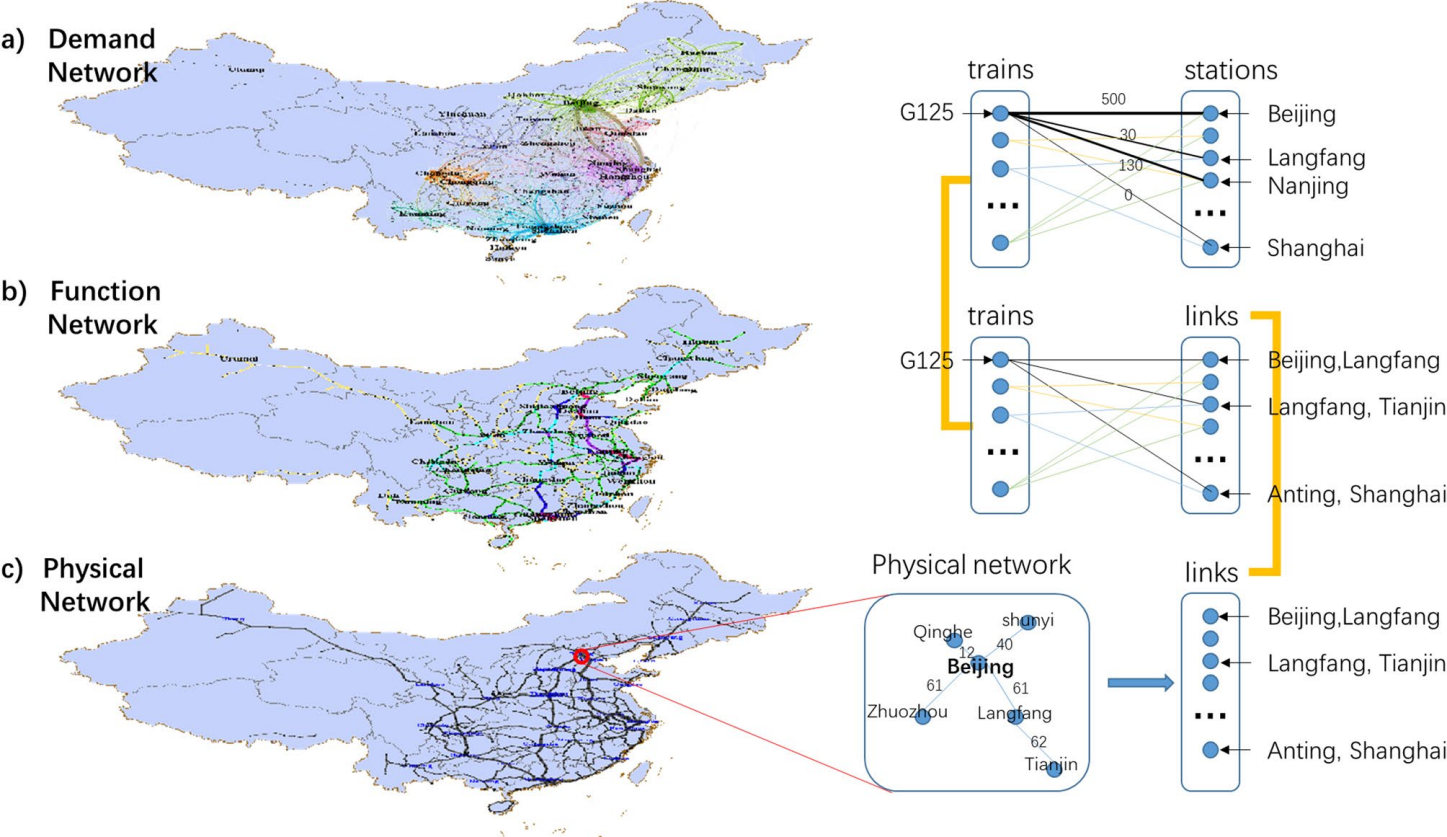
Three-layer network model of HSR. The network visualization was generated using Gephi (version 0.9.2, https://gephi.org).

Physical Network is mainly used to express the location of stations and the connection relationship between stations, and the line in Physical Network is the target to be attacked. Demand Network is mainly used to estimate the number of passengers on each train based on the train schedule. Of course, if the information of all passengers’ tickets can be obtained, it could be used as the accurate data in Demand Network. Unfortunately, the ticket information is confidential in China. Apparently, the physical and demand networks are independent of each other, because the construction of Physical Network only needs the map of HSR, while the construction of Demand Network only has the information of train schedule as input data.

Function Network acts as the bridge network to connect Physical Network and Demand Network. Demand Network figures out the number of passengers on each train, Physical Network describes the connection relationship of stations, and our goal is to figure out the quantity of trains and passengers on each line. Hence, the key to combine Physical Network and Demand Network is construct a network that connects the trains and railway lines. As shown in Fig. 2, the trains are from the Demand Network and the lines are from Physical Network. The objective of Function Network is to find whether a train passing through a specific line, and the difficulty of constructing Function Network is that the schedule of a train usually does not record all the stations that a train passes through, but only the few stations it stops at, which means the connection based on schedule is not the lines in Physical Network. Hence, an inference algorithm is proposed in this paper to complement the omitted stations.

#### Physical network

As shown in Fig. 2(c), The Physical Network G_*P*_ (*S*, *L*_*P*_, *D*) is a topological network based on the stations and lines of high-speed railway network, which is mainly used to observe the location of stations and the distance between stations.

The nodes *S* in Physical Network denote the note set of stations in the HSR, and the lines *L*_*P*_ denote the edge set of railway lines, the weight *D* denotes the distance between two stations (physical length of each line). To construct the Physical Network, information on stations, railway lines, and inter-station distances can be directly obtained from publicly available railway maps and official infrastructure data.

#### Function network

As shown in Fig. 2(b), The Function Network *G*_*F*_ (*T*, *L*_*F*_, *E*) is a bipartite graph, which shows the relationship between trains and the lines. In Function Network, the nodes consist of the set of trains T and line name nodes *L*_*F*_, and the edge *E* represents the connection between trains and lines. The line name nodes *L*_*F*_ corresponds to the lines in physical network one by one. In other words, to establish the relationship between physical network and function network, Edges in the physical network are mapped to each line name node in the function network. For example, there is a line connects Beijing and Langfang in physical network, there would be a node named Beijing, Langfang in function network.

The reason to construct Function Network is to find the relationship between trains and lines. As shown in Fig. 2(b), train G125 is a train from Beijing to Shanghai, its schedule is Beijing – Langfang – Shanghai. It is known that there is not a direct line connecting Langfang and Shanghai in the Physical Network. Actually, there are many lines from Langfang to Shanghai which train G125 must pass through, the objective of Function Network is to find the omitted lines for each train and connect them with edge *e*. Based on the research, G125 would connect with 31 lines name nodes, and any of them is attacked would lead train G125 could not reach its terminal.

Obviously, the degree of the line name node represents the number of trains passing through the line. In the Function Network, when a line is damaged, the trains connecting to the line name nodes are the affected trains, not only the amounts, but also the specific train number is determined, which gives us a chance to analyze the demand of impacted passengers.

The difficulty of constructing a Function Network is to determine the trains’ path from one stop to the next stop. In this paper, an inference algorithm is proposed to complement the omitted stations from one stop to the next stop. For any two adjacent stops in a train schedule, the shortest path between the two stops is chosen as the train’s path. For example, train G125 stops at Beijing, Langfang and Shanghai, the inference will investigate the shortest path from Beijing to Langfang, then Langfang to Shanghai. If a train has *n* stops, the Dijkstra algorithm will be applied *n* − 1 times to form the complete path of the train to connect the train with every line it passes through.

**Figure:**
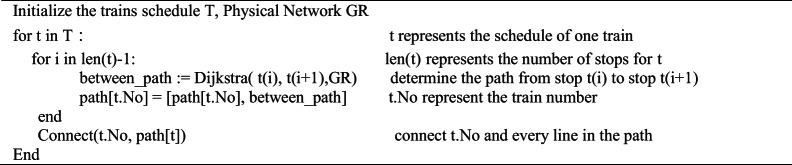

The Function Network constructed in this study should be understood as a timetable-driven baseline service layer rather than a purely static relational graph. It projects how scheduled train services utilize the underlying physical infrastructure and provides the necessary mapping from infrastructure sections to train services and associated passenger demand. Importantly, disruption analysis in this framework is performed in a scenario-based manner: when a physical line segment becomes unavailable, the corresponding functional connections are disabled, and trains operating on the disrupted segment are treated as unable to continue their scheduled services through the affected area. This operation identifies the affected train set and the corresponding disrupted OD passenger group, which then serves as the input to the PTS. The PTS represents traffic management primarily through passenger rerouting/transfer accommodation within the remaining network, evaluating whether disrupted passengers can still complete their intended journeys via feasible direct or one-transfer alternatives, and quantifying recoverable passenger volume and effective passenger loss.

Other operational measures such as short-turning, delaying, or real-time timetable rescheduling are not explicitly modeled because they require detailed circulation, crew, rolling-stock, and dispatching constraints that are not available at the nationwide level; moreover, incorporating such microscopic dynamics would extend beyond the scope of network-level vulnerability assessment. Nevertheless, their aggregate consequences are partially reflected in our disruption representation, as treating trains on disrupted segments as unavailable corresponds to cancellations or forced short-turning, while reduced functional connectivity naturally constrains rerouting opportunities.

#### Demand network

Demand Network G_*D*_ (*T*, *S*, *V*) is mainly used to illustrate the number of passengers on each train at each stop, so its bipartite graph composed of the set of trains *T* and the set of stations *S*, as shown in Fig. 2. The edge between the train node and the station node denotes that the train stops at the station and the weight of the edge *V* represents the number of passengers boarding at the station. As shown in Fig. 2(a), the train G125 stops at Beijing station, and there are 500 passengers get on, then train node G125 would connect station node Beijing, and the weight of the edge is 500.

### Passenger service-oriented HSR vulnerability assessment framework

This study develops a passenger service-oriented vulnerability assessment framework for high-speed rail infrastructure networks. First, a definition of HSR vulnerability is proposed from the perspective of passenger service. Subsequently, a Passenger Demand Estimation Model (PDEM) is constructed to forecast passenger flows, followed by the development of a Passenger Transfer Simulation (PTS) model to simulate passengers’ willingness to transfer in the event of service disruptions. Finally, the HSR infrastructure network vulnerability is assessed by integrating the estimated passenger demand and transfer willingness.

It is worth noting that in this article, HSR vulnerability is evaluated from a service-capacity-oriented perspective, with the primary objective of assessing how effectively the railway system can satisfy aggregate passenger demand under disruption scenarios. At the national scale, passenger demand is therefore represented in a macroscopic and system-oriented manner rather than through detailed individual choice modeling.

Specifically, passenger demand is estimated using the proposed Passenger Demand Estimation Method (PDEM), which derives passenger flow directly from the nationwide train timetable. In China, the national train schedule is designed and updated based on actual railway passenger traffic and relevant regulations. Its stopping patterns and service frequencies are intended to reflect the planned capacity of the system to meet passenger travel demands. When aggregated to the national level, the estimated daily passenger volume obtained by PDEM is highly consistent with officially published daily ridership statistics, supporting the validity of this simplified demand representation for large-scale analysis.

Although multi-criteria behavioral models, such as logit formulations, are indispensable for analyzing individual passenger choices, they require detailed passenger trajectory, ticketing, or socio-economic data. Such data are currently not accessible in China due to privacy protection and regulatory constraints. In contrast, the timetable-based approach adopted in this study provides a data-feasible, computationally efficient, and scalable solution for nationwide HSR vulnerability assessment.

Most importantly, the aim of this study is to identify global critical nodes and links and to analyze how local infrastructure disruptions propagate through the entire multi-layer railway network. Accordingly, the proposed framework emphasizes macro-level network behavior rather than micro-level individual decision-making. For this purpose, a simplified yet consistent representation of passenger demand is sufficient to support the research objectives while enabling the method to scale to the full national HSR network.

#### Vulnerability assessment model of HSR

The vulnerability of railway lines refers to the sensitivity of the HSR infrastructure network to performance degradation following line disruptions or failures. Given that the core mission of high-speed railway systems is to fulfill passenger travel demand, this study employs a service-capacity-oriented vulnerability indicator in which passenger flow serves as the primary evaluation metric. Rather than relying on traditional topological measures or multi-criteria behavioral indicators, this study evaluates vulnerability based on the loss of effective passenger-carrying capacity, which directly reflects the operational consequences experienced by the system under disruption scenarios.

This indicator is selected for three reasons. First, it is conceptually aligned with the service-oriented framework of this research. As the railway system fundamentally exists to provide transportation services, measuring the reduction in service capacity offers a more realistic representation of system performance deterioration than structural connectivity metrics alone. Second, multi-criteria indicators, such as lost passengers, detour distance, and increased travel time, require detailed passenger trajectory data, which are not accessible in China due to privacy restrictions and data protection regulations. In contrast, the proposed indicator can be calculated using publicly obtainable scheduling and infrastructure data, making it suitable for nationwide, large-scale HSR network assessment. Third, although the indicator is single-dimensional, it implicitly captures a wide range of system-level impacts caused by disruptions, such as train cancellations, loss of connectivity, reduced rerouting flexibility, and bottleneck formation. This is because the indicator is derived from the three-layer coupled network constructed in this study, which integrates the physical infrastructure layer, the functional service layer, and the passenger demand layer. As a result, the service-capacity loss metric inherently incorporates information from multiple layers of the network, reflecting the combined effects of structural constraints, operational adjustments, and demand redistribution. Consequently, the proposed indicator offers a computationally efficient yet comprehensive representation of the behavioral and operational consequences of disruptions across the multi-layer high-speed railway system.

In summary, this study constructs a vulnerability assessment index for high-speed rail systems, using passenger flow as the primary evaluation indicator. The service-oriented vulnerability index is defined as follows (Eq. 1):

1 $$\begin{array}{*{20}{c}} {RV=\frac{{{P^{loss}}}}{{{P^{normal}}}}} \end{array}{\mathrm{~}}$$

Where, *RV* represents the vulnerability of HSR. ${P^{loss}}$ denotes the loss of performance caused by the failure of lines. In this paper, it is represented by the number of passengers who could not reach their destinations because of the line failure. ${P^{normal}}$ represent the number of passengers who complete their trips in normal operation.

To calculate ${P^{loss}}$ from the failure of lines, the three-layer network model is established to figure out the affected passengers ${P^{affected}}$ and the transfer passengers ${P^{transfer}}$. Loss passenger flow ${P^{loss}}$ and transfer passenger flow ${P^{transfer}}$ together constitute the affected passenger flow ${P^{affected}}$, because some of the affected passengers could choose other trains to complete their trip. So ${P^{loss}}$ could be calculated by Eq. (2).

2 $$\begin{array}{*{20}{c}} {{P^{loss}}={P^{affected}} - {P^{transfer}}} \end{array}$$

Through the above analysis, the key step to evaluate line vulnerability is to determine ${P^{affected}}$ and ${P^{transfer}}$. Therefore, to accurately estimate the number of affected passengers, this study proposes a novel and precise model of passenger demand along with a corresponding passenger transfer strategy.

#### Passenger demand estimation method (PDEM)

It’s easy to construct the Demand Network from the ticket information, but the data is confidential and unclosed in China. The only data can be obtained is the annual number of passengers. In traditional research, the p-space network is usually used to estimate the passenger flow. The core objective of this study is to evaluate the service-providing capacity of the railway network under disruption scenarios. Our methodological framework is grounded in the operational information embedded in the national railway timetable. According to the Train Schedule Compilation and Management Regulation (Document No. Tieyun [2008] 206), train stopping patterns and service frequencies must be planned in accordance with the spatial distribution and intensity of passenger demand. Based on these regulatory principles, we extract key operational parameters from the timetable, such as line-level service frequency and station-level stopping frequency, to infer the passenger transportation demand that the network is designed to accommodate under normal operating conditions.

In contrast to studies that emphasize individual passenger behavioral characteristics, the present study adopts the official timetable as the analytical foundation. The timetable is assumed to represent a standardized operational plan generated under the regulation’s “demand-matching” principle. Accordingly, given that the regulation explicitly requires train stopping frequencies to align with station-level demand intensity, a higher number of scheduled train stoppings at a station generally indicates a higher underlying level of passenger transportation demand. This provides a practical and data-consistent basis for the demand estimation employed in this study.

For the reasons outlined above, the Passenger Demand Estimation Method (PDEM) adopts a simplified, timetable-based assumption regarding passenger distribution along each train’s route. The model assumes that, between the departure and terminal stations, the total number of passengers on a train remains approximately constant, with major boarding concentrated at the origin and major alighting at the destination. Accordingly, boarding and alighting flows at intermediate stations are treated as roughly balanced.

Building on this timetable-based demand inference, the Passenger Demand Estimation Method (PDEM) adopts an operationally grounded assumption regarding passenger distribution along each train’s route. In China’s high-speed railway system, train capacity planning is fundamentally based on the 8-car standard formation, with demand elasticity accommodated mainly through adjustments in service frequency rather than through substantial changes in individual train loading. Although passenger flow varies throughout the day, the network operates a fixed nighttime maintenance window during which regular services are not run except during rare peak travel periods such as the Spring Festival travel rush. This allows the timetable to smooth out time fluctuations and reflect the overall service capacity of the system.

This assumption is also consistent with empirical HSR travel patterns, where long-distance OD flows dominate and most passengers board at origins and alight at terminals, leaving intermediate boarding and alighting largely balanced at the aggregate level. Under these operational characteristics, the total number of passengers on board can reasonably be treated as approximately constant between the origin and terminal stations. Given the unavailability of individual-level passenger data in China, this abstraction offers a practical, data-feasible, and operationally consistent representation that aligns well with the service-capacity perspective of this study and supports system-level vulnerability assessment under disruption scenarios.

The capacity of a train is C, and the proportion of passengers at the departure station is assumed as p. Passenger boarding volumes at a station are strongly influenced by the number of trains that stop there. In practice, stations with higher stopping frequencies typically correspond to major urban centers or transfer hubs, where passenger alighting, boarding, and transferring activities are substantially more intensive. Because the high-speed rail timetable is designed to match stopping patterns with local demand intensity, the stopping frequency naturally encapsulates the relative passenger activity level at each station. Accordingly, even though individual passenger trajectory data are not available, stations that function as major transfer hubs are structurally recognized in our model through their higher stopping frequencies.

In this study, the number of passengers boarding at a stopping station is represented using a piecewise function. Specifically, when the number of trains stopping at a station is very small, the station is assigned a minimum passenger flow ${V}_{min}$. Conversely, when the stopping frequency is very high, the station is assigned a maximum passenger flow${V}_{max}$. For stations where the number of stopping trains lies between these two extremes, a linear function is used to estimate the boarding or alighting volume as a function of the number of stopping trains.

Therefore, for a train *t*, the number of passengers at station *S* can be expressed as Eq. (3):

3 $$\begin{array}{*{20}{c}} {{V_{s,t}}=~\left\{ {\begin{array}{*{20}{c}} {C{\mathrm{*}}p}&{t \in H\left( {{T_S}} \right)} \\ {{V_{min}}}&{f\left( {\left| {{\mathrm{I}}\left( {{T_S}} \right)} \right|} \right) \leqslant {V_{min}},{\mathrm{~}}t \in {\mathrm{I}}\left( {{T_S}} \right)} \\ {\begin{array}{*{20}{c}} {f\left( {\left| {{\mathrm{I}}\left( {{T_S}} \right)} \right|} \right){\mathrm{~}}} \\ {{V_{max}}} \\ 0 \end{array}}&{\begin{array}{*{20}{c}} {{V_{min}}<f(\left| {{\mathrm{I}}\left( {{T_S}} \right)} \right| \leqslant {V_{max}},t \in {\mathrm{I}}\left( {{T_S}} \right)} \\ {f\left( {\left| {{\mathrm{I}}\left( {{T_S}} \right)} \right|} \right)>{V_{max}},t \in {\mathrm{I}}\left( {{T_S}} \right)} \\ {t \in T\left( {{T_S}} \right)} \end{array}} \end{array}} \right.} \end{array}$$

Where, ${T_S}$ is the set of all trains stop at station *S*, and $\left| {{T_S}} \right|$ represents the number of trains stop at stations *S*. *C* is train capacity. in this article. H(${T_S}$) represents the set of trains departing from station *S*. T(${T_S}$) represents the set of trains terminating at station S. I(${T_S}$) represents the set of trains stopping at station S as an intermediate station, which can be expressed as ${\mathrm{I}}\left( {{T_S}} \right)={T_S} - {\mathrm{T}}\left( {{T_S}} \right)$.

The number of passengers at station *S* can be expressed as Eq. (4):

4 $$\begin{array}{*{20}{c}} {{V_S}=\mathop \sum \limits_{{t\epsilon Ts}} {V_{s,t}}} \end{array}$$

By constructing the value ${V_S}$ for all stations, this study constructs a complete demand network.

The number of passengers of train *t* can be expressed as Eq. (5):

5 $$\begin{array}{*{20}{c}} {{V_t}=\mathop \sum \limits_{{S\epsilon St}} {V_{s,t}}} \end{array}$$

Where, ${S_t}$ stands for the stops in the schedule of train *t*.

From formula (4) and formula (5), the total passenger volume under disruption and normal operation can be calculated as:

6 $$\begin{array}{*{20}{c}} {{P^{affected}}=\mathop \sum \limits_{{t=1}}^{n} \mathop \sum \limits_{{S=1}}^{m} {V_{s,t}}} \end{array}$$

7 $$\begin{array}{*{20}{c}} {{P^{normal}}=\mathop \sum \limits_{{t=1}}^{i} \mathop \sum \limits_{{S=1}}^{j} {V_{s,t}}} \end{array}$$

Equation (6) is used to calculate the total number of passengers under the affected state, while Eq. (7) represents the total passenger volume under normal operation. By adjusting the parameter of the estimation method, the estimation of passenger flow can be very close to the disclosed data.

#### Passengers Transfer Strategy (PTS)

The most difficult part of evaluating the vulnerability of a large-scale HSR network is to estimate the number of transferable passengers. When a railway line is disrupted, hundreds of trains may be affected, and the disrupted passenger demand must be reassigned by identifying feasible alternative services from thousands of remaining trains. This requires systematically determining available transfer trains, potential transfer stations, and corresponding seat capacities.

Under disruption scenarios, this study represents traffic management primarily through passenger rerouting, which provides a system-level and methodologically consistent mechanism to evaluate whether passengers affected by line disruptions can still complete their intended journeys. When a railway line becomes unavailable, all trains operating on the disrupted segment in the Functional Network are treated as unable to continue their scheduled services. Accordingly, passengers traveling between the departure and terminal stations of these trains are considered unable to complete their planned OD trips and are identified as the primary group of affected passengers.

In HSR operations, long-distance OD passengers typically account for the majority of on-board demand. The PTS is therefore designed to evaluate rerouting possibilities for these passengers, who are most directly impacted when a long-distance service is interrupted. Based on nationwide timetable information, the PTS first examines whether alternative trains departing from the same origin station can directly reach the passenger’s destination. If no such direct service exists, the intermediate stops of these origin-departing trains are treated as potential transfer stations, and the model further evaluates the feasibility of one-transfer routes within the remaining network. Through this structured procedure, the PTS determines whether disrupted passengers can be reassigned to alternative services, and the number of passengers who cannot be accommodated is quantified as effective passenger loss due to the disruption.

Other traffic management measures, such as short-turning, delaying, or real-time rescheduling, are not explicitly modeled in this study. These measures involve detailed operational decision-making processes that depend on train circulations, crew schedules, rolling-stock constraints, and real-time operational margins, and they are typically implemented as short-term dispatching strategies to stabilize local operations. In contrast, the objective of this research is to evaluate the system-wide degradation of passenger service capacity caused by infrastructure disruptions. Explicitly modeling such microscopic operational dynamics would extend beyond the scope of network-level vulnerability analysis and introduce additional behavioral and scheduling uncertainties that cannot be robustly addressed within the present framework.

Nevertheless, the aggregate effects of these operational measures are implicitly captured in the modeling approach. Treating all trains operating on the disrupted segment as unavailable reflects the operational consequences of cancellations or forced short-turning, while the resulting reduction in functional network connectivity naturally represents the decreased rerouting flexibility that typically arises under delays or timetable adjustments.

To operationalize the PTS described above, this study makes a simplifying yet computationally tractable assumption regarding passenger travel alternatives under disruption scenarios. Specifically, affected passengers are assumed to consider only two types of feasible alternatives:

1. direct transfers, in which passengers are reassigned to trains that can reach their destination without intermediate transfers;
2. one-transfer schemes, in which passengers complete their journeys via a single intermediate transfer station.

Under this assumption, identifying feasible transfer options requires searching across the infrastructure network, train services, station stops, and passenger OD relations. If a simple sequential search algorithm were applied, the computational complexity for enumerating all direct transfer options would be on the order of $$O\left( {m*n*p*q} \right),$$ whereas the complexity for enumerating all one-transfer options would increase to $$O\left( {m*n*p*q*p*q} \right).$$

Here, *m* denotes the total number of track segments (lines) in the HSR infrastructure network, *n* denotes the number of trains operating on a given line, *p* denotes the number of trains stopping at a given station, and *q* denotes the number of stations served by each train. This rapid growth in computational complexity highlights the necessity of carefully designed search strategies to ensure scalability when analyzing nationwide HSR networks.

For the direct transfer case, the algorithm needs to check the compatibility between all links *(m)*, the trains running on them *(n)*, the trains stopping at a transfer-related station *(p)*, and all possible stopping stations of those trains *(q)*. Therefore, the total search effort is proportional to the product $$O\left( {m*n*p*q} \right)$$.

For the one-transfer case, the algorithm must additionally evaluate the combinations of two consecutive steps (origin → transfer → destination). This results in an additional multiplicative factor of $$\left( {p*q} \right)$$ for the second step, leading to the worst-case complexity $$O\left( {m*n*p*q*p*q} \right)$$.

For a large-scale network, there may be tho*u*sands of lines and hundreds of trains passing through a line, hundreds of trains stop at a station, and dozens of stations a train can reach, which causes a huge time complexity.

In order to quickly generate transfer scheme for a large number of passengers, the reachable matrix is introduced to reduce the time complexity, which reduce the time complexity of transfer with two trains from $$O\left( {m*n*p*q*p*q} \right)$$ to $$O\left( {m*n^{\prime}*p*q} \right)$$. The reachable matrix is a $${\mathrm{n}} \times {\mathrm{n}}$$ matrix, $${{{\rm n}^{\prime}~}}$$represents the number of stations. For the construction of the city reachability matrix, we first obtain the stop set $${{\mathrm{S}}_{\mathrm{t}}}$$ for each train t, If two stations $${{\mathrm{s}}_{\mathrm{i}}}{\mathrm{~}}$$and $${{\mathrm{s}}_{\mathrm{j}}}$$ both belong to $${S_t}$$,i.e. $${s_i},{s_j} \in {S_t}$$, then the entry $$M~\left( {{s_i},~{s_j}} \right)$$ in the city reachable matrix is assigned the identifier of train t; otherwise, the corresponding entry remains empty. By traversing all trains in the timetable and repeating this procedure, the complete city reachability matrix *M* is generated. It records all trains from station *i* to station *j*, as shown in Table 2. $$M~\left( {{s_1},~{s_2}} \right)~$$means the passengers could reach $${{\mathrm{s}}_2}$$ from $${{\mathrm{s}}_1}$$ by train G1001 or G1003, and the computational complexity is $$O(\alpha *\beta *\left( {\beta - 1} \right)$$), Where $${\mathrm{\boldsymbol{\upalpha}}}$$ represents the number of trains in the timetable, and $${\mathrm{\boldsymbol{\upbeta}}}$$ represents the number of stops of the trains in the timetable.

**Table 2:** The reachable matrix.

Station	s1	s2	s3	…
s1	/	G1001, G1003	G1001, G1005	…
s2	G1002	/	G1004	…
s3	G1004	G1002	/	…
…	…	…	…	…

The above complexity analysis indicates that, without structural optimization, the enumeration of feasible passenger transfer schemes under disruption scenarios would lead to a rapid combinatorial explosion, particularly for the one-transfer case. In large-scale HSR networks, where the number of line segments, trains, station stops, and feasible OD combinations is high, a naive sequential search strategy would make nationwide vulnerability assessment computationally impractical.

To verify that the introduction of the reachable matrix effectively mitigates this complexity growth in practice, a computational time experiment was conducted. The experiment compares the proposed matrix-based PTS with a naive sequential enumeration implementation that directly follows the search logic implied by the worst-case complexities $$O\left( {m*n*p*q} \right)$$and $$O\left( {m*n*p*q*p*q} \right)$$.

The experiment focuses on the 50 busiest railway sections, selected according to the number of trains operating on each section, which represent the most computationally demanding scenarios. For each section, passenger transfer evaluation was performed under identical disruption conditions using both implementations, and the corresponding runtimes were recorded.

The results demonstrate a substantial reduction in computational cost achieved by the reachable-matrix-based PTS. On average, the naive sequential implementation requires approximately 1.12 s per section (median: 0.86 s), whereas the matrix-based implementation reduces the average runtime to about $3.0 \times {10^{ - 4}}$s per section (median: $2.5 \times {10^{ - 4}}$s). This corresponds to an average acceleration ratio of approximately $3.77 \times {10^3}$, with speedups ranging from about $1.6 \times {10^3}$to $5.7 \times {10^3}$across different sections.

These results empirically confirm that the reachable matrix effectively transforms multi-level nested search processes into index-based queries, thereby substantially reducing the practical computational burden of passenger transfer enumeration. By bridging the gap between theoretical scalability and practical applicability, the proposed PTS enables rapid vulnerability screening and large-scale disruption scenario analysis for nationwide high-speed railway networks.

When a railway line is disrupted, all trains operating on the affected segment in the Functional Network are regarded as unable to continue their scheduled services, and the passengers traveling between their origin and terminal stations are therefore unable to complete their planned trips. In high-speed railway operations, long-distance OD passengers those traveling directly between the departure and terminal stations typically constitute the majority of on-board demand. Accordingly, the PTS in this study is designed specifically for these OD passengers, assessing whether they can still reach their destinations through the remaining network connectivity. Using timetable information, the PTS first examines whether alternative trains departing from the same origin can directly reach the destination; if not, the stops of these origin-departing trains are treated as potential transfer stations from which feasible one-transfer routes are explored. Through this rerouting process, the model estimates how many affected passengers can be reassigned to the residual network, while implicitly reflecting the service loss caused by train cancellations on the disrupted segment.

First, for all trains that operate on the disrupted segment *l*, we form an affected set ${T_l}$ which is called the affected train set. Furthermore, each train has a $V_{t}^{{transfer}}$ indicating the number of passengers that can transfer.

For each train $t \in {T_l}$, let its origin and destination be denoted as ${O_t}{\mathrm{~}}$and ${D_t}$. The OD passenger volume between ${O_t}$ and ${D_t}$ cannot exceed the number of passengers boarding at the origin station, i.e., $Cp$. The transferable passenger volume on train *t* is initialized as:

$$V_{t}^{{transfer}}=0$$

Let $T\left( {{O_t}} \right)$ denote the set of trains stopping at station ${O_t}$. For each train ${t_{{O_t}}} \in T\left( {{O_t}} \right)$, let $S\left( {{t_{{O_t}}}} \right)$ denote the set of stations not yet served by this train.

If:

$${D_t} \in S\left( {{t_{{O_t}}}} \right)$$

then train ${t_{{O_t}}}$can directly reach ${D_t}$, and we update:

$$V_{t}^{{transfer}}=\mathop \sum \limits_{{{t_{{O_t}}} \in T\left( {{O_t}} \right)}} C\left( {1 - p} \right)$$

This indicates that train ${t_{{O_t}}}$can provide direct-transfer service for up to $C\left( {1 - p} \right)$ passengers originally traveling on train *t*.

If none of the trains departing from ${O_t}$ can directly reach ${D_t}$, we consider one-transfer routes. For each train ${t_{{O_t}}} \in T\left( {{O_t}} \right)$, let the stations it can reach be denoted as $S\left( {{t_{{O_t}}}} \right)$.

For each potential transfer station:

$${s^{transfer}} \in S\left( {{t_{{O_t}}}} \right)$$

We examine the trains $T\left( {{s^{transfer}}} \right)$departing from ${s^{transfer}}$ to determine whether they can reach ${D_t}$.

If a train ${t_{{s^{transfer}}}} \in T\left( {{s^{transfer}}} \right)$. can reach ${D_t}$, its remaining capacity is assigned to accommodate transferring passengers. We update:

$$V_{t}^{{transfer}}=\mathop \sum \limits_{{~{t_{{s^{transfer}}}} \in T\left( {{s^{transfer}}} \right)}} C\left( {1 - p} \right)$$

The corresponding transfer route is:

$${t_{{O_t}}} \to {s^{transfer}} \to {t_{{s^{transfer}}}}.$$

In summary, the total number of transfer passengers ${P^{transfer}}$ whose travel needs can be met when an interruption occurs can be obtained from the following formula:

$${P^{transfer}}=\mathop \sum \limits_{{t \in {T_l}}} V_{t}^{{transfer}}$$

And the algorithm is shown in the following:

**Figure:**
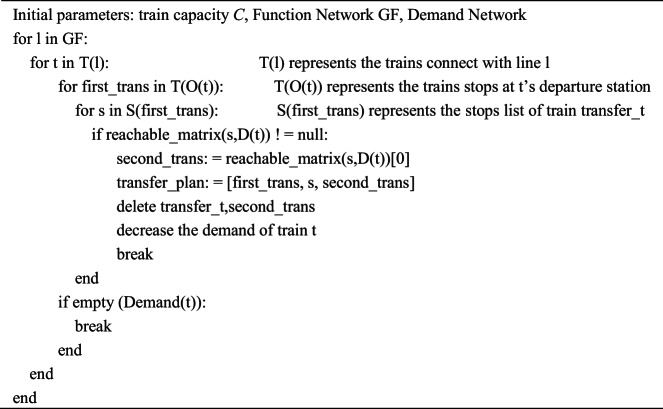

#### Passenger service-oriented vulnerability mode

Above, the passenger service-oriented vulnerability of HSR is as Eq. (8):

8 $$\begin{array}{*{20}{c}} {RV=\frac{{\mathop \sum \nolimits_{{t=1}}^{n} \mathop \sum \nolimits_{{S=1}}^{m} {V_{s,t}} - \mathop \sum \nolimits_{{t \in {T_l}}} V_{t}^{{transfer}}}}{{\mathop \sum \nolimits_{{t=1}}^{i} \mathop \sum \nolimits_{{S=1}}^{j} {V_{s,t}}}}} \end{array}$$

And this paper also focus on analyzing the vulnerability of a single railway line. For any railway line *l* in Physical Network, the vulnerability of the line *l* can be expressed as Eq. (9).

9 $$\begin{array}{*{20}{c}} {R{V_l}=\frac{{P_{l}^{{affected}} - P_{l}^{{transfer}}}}{{P_{l}^{{normal}}}}} \end{array}{\mathrm{~}}$$

### Sensitivity analysis

To evaluate the robustness of the proposed service-oriented vulnerability assessment, a sensitivity analysis is conducted on several core parameters involved in the Passenger Demand Estimation Method (PDEM). Let $\theta$ denote a generic model parameter and ${\theta _0}$ its baseline value used in the main analysis. For each parameter, we examine how perturbations around ${\theta _0}$ affect both the magnitude and the spatial pattern of the vulnerability results.

The vulnerability of line *l* under a given parameter setting $\theta$ is defined as:

$$R{V_l}\left( \theta \right)=1 - \frac{{P_{l}^{{transfer}}\left( \theta \right)}}{{P_{l}^{{normal}}\left( \theta \right)}}$$

Where $P_{l}^{{transfer}}\left( \theta \right)$and $P_{l}^{{normal}}\left( \theta \right)$ denote the number of passengers who can successfully complete their trips before and after the disruption of line *l*, respectively, as evaluated by the coupled network, PDEM, and PTS. A larger value of $R{V_l}\left( \theta \right)$ indicates a higher vulnerability of line *l* from the perspective of passenger service capacity.

For each parameter $\theta$, a one-factor-at-a-time perturbation scheme is adopted. Specifically, $\theta$ is varied within a predefined range around ${\theta _0}$ while all other parameters are kept at their baseline values. The following parameters are examined:

1. the boarding proportion at the origin station *p*, which controls the distribution of OD passenger volume in PDEM;
2. the lower and upper bounds of station-level passenger load ${V_{{\mathrm{min}}}}$ and ${V_{{\mathrm{max}}}}$, which determine how stopping frequency is mapped to demand intensity.

At the network level, the average vulnerability under parameter $\theta$is defined as:

$$RV\left( \theta \right)=\frac{1}{{\mid L\mid }}\mathop \sum \limits_{{l \in L}} R{V_l}\left( \theta \right)$$

Where *L* is the set of all lines in the high-speed railway infrastructure network. The relative sensitivity of the network-wide vulnerability to parameter $\theta$ is then quantified by

$${S_\theta }=\frac{{\mid RV\left( \theta \right) - RV\left( {{\theta _0}} \right)\mid }}{{RV\left( {{\theta _0}} \right)}}.$$

A small value of ${S_\theta }$ indicates that the overall level of vulnerability is stable with respect to perturbations of $\theta$.

In addition to magnitude changes, the stability of the spatial pattern of vulnerability is evaluated by comparing the ranking of lines under the baseline and perturbed parameter settings. Let ${\mathrm{Rank}}\left( {{\theta _0}} \right)$ and ${\mathrm{Rank}}\left( \theta \right)$ denote the ranking vectors of $R{V_l}\left( {{\theta _0}} \right)$and $R{V_l}\left( \theta \right)$ over all lines $l \in L$. The rank stability is measured by the Spearman rank correlation coefficient.

$${\rho _\theta }={\mathrm{Spearman}}\left( {{\mathrm{Rank}}\left( {{\theta _0}} \right),{\mathrm{Rank}}\left( \theta \right)} \right)$$

Values of ${\rho _\theta }$ close to 1 indicate that the identification and ordering of critical lines are highly robust to changes in $\theta$.

By jointly examining the relative sensitivity index ${S_\theta }$and the rank correlation ${\rho _\theta }$for all tested parameters $\left( {p,{\mathrm{~}}{V_{{\mathrm{min}}}},{V_{{\mathrm{max}}}}} \right)$, the sensitivity analysis provides a systematic assessment of how parameter uncertainty affects the service-oriented vulnerability results, and demonstrates that the proposed framework remains stable within a reasonable perturbation range of the key modeling assumptions.

## Case study

### Data description

This paper collects high-speed railway train operation data on a certain day in December 2025 from the Railway Customer Service Center of China (https://www.12306.cn), including the train number and stopover information. A total of 77,603 stops were recorded, involving 8,726 trains, with an average of 9 stops per train. The infrastructure Network data is obtained from Baidu Map (https://api.map.baidu.com/lbsapi/getpoint/index.html?qq-pf-to=pcqq.discussion).

### Physical network analysis

**Fig. 3:**
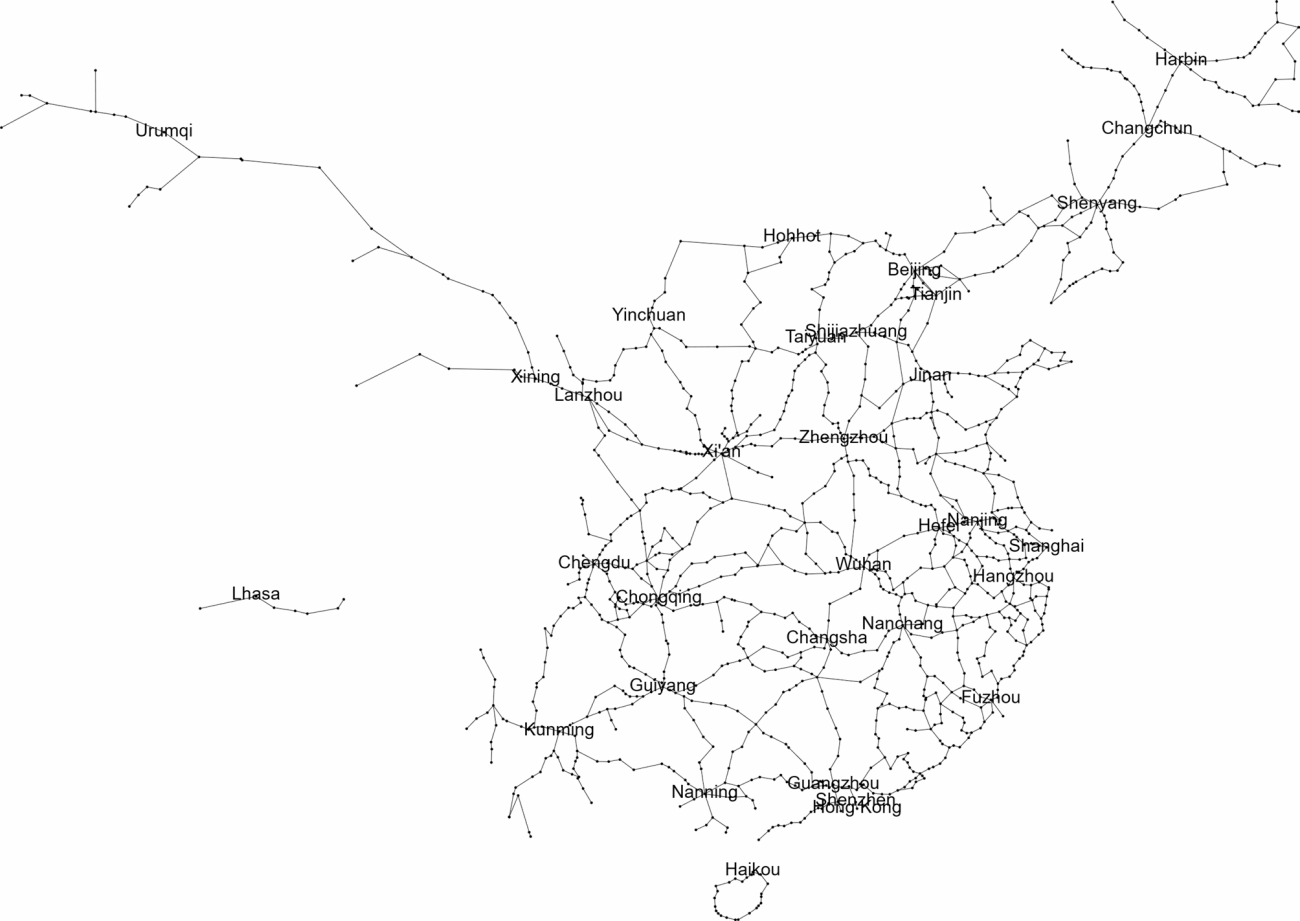
Physical network of HSR. The network visualization was generated using Gephi (version 0.9.2, https://gephi.org).

To study the network characteristics of the railway infrastructure network, this study merges some stations and lines in the city. Therefore, 277 stations are merged with nearby stations, and finally an undirected weighted graph is formed with 1173 stations as nodes and 1341 railway sections as edges. The length of the railway section is approximated by the straight-line distance between two stations. Based on the above network information, G_*P*_ (*S*, *L*_*P*_, *D*) is constructed as shown in Fig. 3.

The configuration is dominated by several metropolitan hubs that emerge as high-degree and high-centrality vertices, and their spatial arrangement gives rise to a multi-center framework that governs the distribution of flows and the prioritization of corridor development. Around these hubs, link patterns form coherent radial structures that connect peripheral regions to the primary network core.

The spatial heterogeneity visible in the figure mirrors the unequal distribution of infrastructure density across the territory. Eastern and central regions contain tightly clustered nodes and high link densities, while the western portion is characterized by sparse spatial embedding. This heterogeneity embodies the constraints imposed by terrain, urbanization and economic specialization, and it produces a structural imbalance that reinforces differences in regional accessibility. Despite these disparities, the global configuration suggests that the system operates with small-world tendencies in which long-range edges and hub-mediated shortcuts significantly reduce effective distances between node pairs. Such properties support high operational efficiency but simultaneously increase the dependence of the entire system on a limited number of strategically positioned intermediaries.Within this architecture, several nodes act as critical inter-regional connectors whose structural roles correspond to elevated betweenness centrality. These nodes support the integration of multiple corridors and facilitate a substantial share of the shortest paths linking geographically distant regions. Their prominence implies that localized disruptions may lead to disproportionate consequences by inducing large-scale fragmentation or by reconfiguring flow distribution across secondary corridors.

Besides critical hub nodes, certain railway line segments often connect high-level or highly central nodes along major railway corridors, thereby serving as indispensable channels for long-distance passenger flows. When these critical line segments are disrupted, the consequences extend beyond simple increases in travel distance. Such disruptions may force passenger flows to detour through alternative routes with lower service capacity or greater geographical constraints, significantly reshaping the overall network path structure.

As a result, these railway line segments constitute key structural dependencies within the network and play a crucial role in the analysis of system vulnerability and passenger transport efficiency.

Although these structural features provide an important foundation for understanding the organization of the railway system, a purely topology-based perspective is insufficient for capturing the dynamics of real-world railway operations. Structural prominence does not always coincide with operational criticality, and sections that appear peripheral in the abstract graph may in practice accommodate substantial traffic volumes or represent indispensable components of regional transport. For this reason, the present study extends beyond the topological characterization of nodes and edges and focuses explicitly on identifying stations and sections that exert disproportionate influence on service capacity. By integrating structural indicators with measures that reflect operational load and functional importance, the analysis aims to identify those elements whose disruption would produce the largest degradation in actual transport capability.

### Function network analysis

Using data from 77,603 train timetable stops and 8,726 high-speed trains, this study constructs a functional network by combining physical network. Within the three-layer network modeling framework, the function network serves as the critical intermediate layer that links the static topology of the physical infrastructure with the dynamic characteristics of operational services. It is constructed by mapping the sequential stopping patterns of train services onto the set of physical nodes and edges, thereby transforming the timetable-driven operational process into a structured representation of functional connectivity. Through this projection, each physical section acquires a functional weight that reflects the cumulative frequency with which it is traversed by train services and each station inherits a load level derived from the aggregated stopping and transfer activities imposed by scheduled operations. In this way, the function network expresses how real operations activate and utilize the underlying infrastructure, revealing a dynamic layer of connectivity that cannot be inferred from the static physical graph alone.

**Fig. 4:**
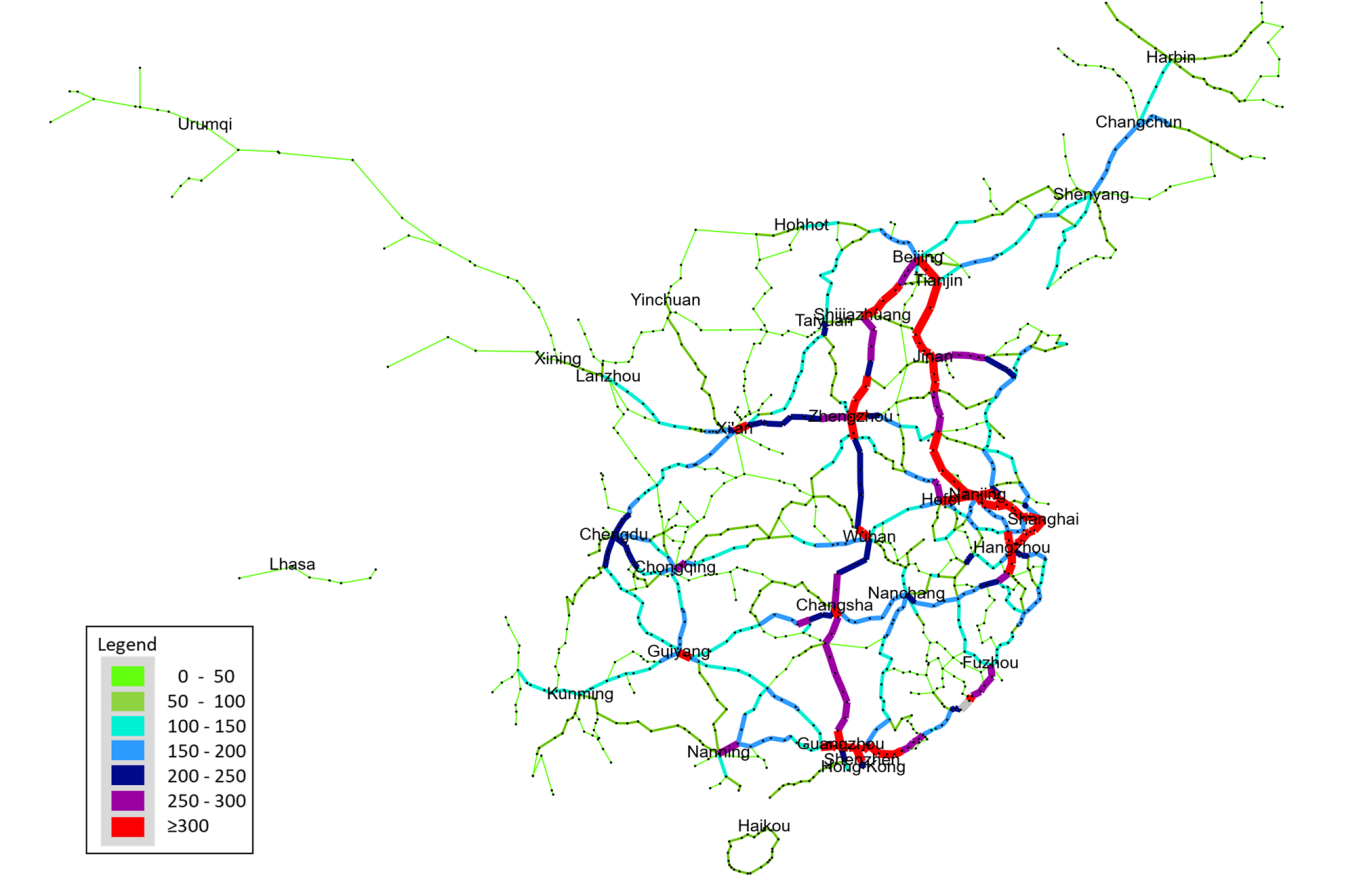
Function network of HSR. The network visualization was generated using Gephi (version 0.9.2, https://gephi.org).

**Fig. 5:**
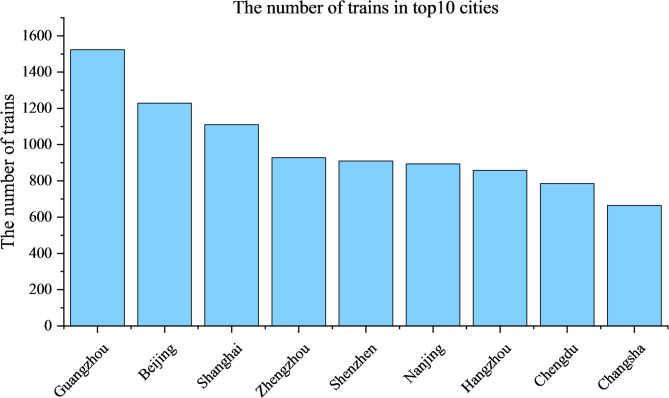
The number of trains in Top 10 cities.

As shown in Fig. 4, It’s obvious that there are two busiest long -distance lines, Beijing-Nanjing-Shanghai line and Beijing-Guangzhou-Shenzhen line. The Beijing-Shanghai line has an average daily traffic flow of more than 250 trains, followed by the Beijing-Guangzhou Line with more than 150 trains. Besides, Zhengzhou-Xi’an Line is quite busy. As shown in Fig. 5, Guangzhou has the most trains stopping there, with 1524 trains, followed by Beijing with 1229 trains. Shanghai ranks third in terms of the number of trains stopping there, with 1110 trains. It is quite evident that due to the considerable heterogeneity in the spatial distribution of functional intensity, certain sections and stations absorb a disproportionate amount of national train traffic. Highly traversed north–south and east–west axes emerge as dominant channels for long-distance mobility, while peripheral or geographically challenging regions exhibit markedly lower functional participation. This uneven allocation of operational load reflects the combined influence of passenger demand, section planning, service frequency scheduling and regional transport roles. Furthermore, it is easy to see that functional centrality does not always align with structural centrality: some structurally ordinary nodes become indispensable in operation due to their role in high-frequency train flow; while some prominent nodes in the topology may have relatively limited operational burdens. Therefore, functional networks provide a more refined description of system behavior by revealing the emergent operational dependencies hidden in the physical layer.

**Fig. 6:**
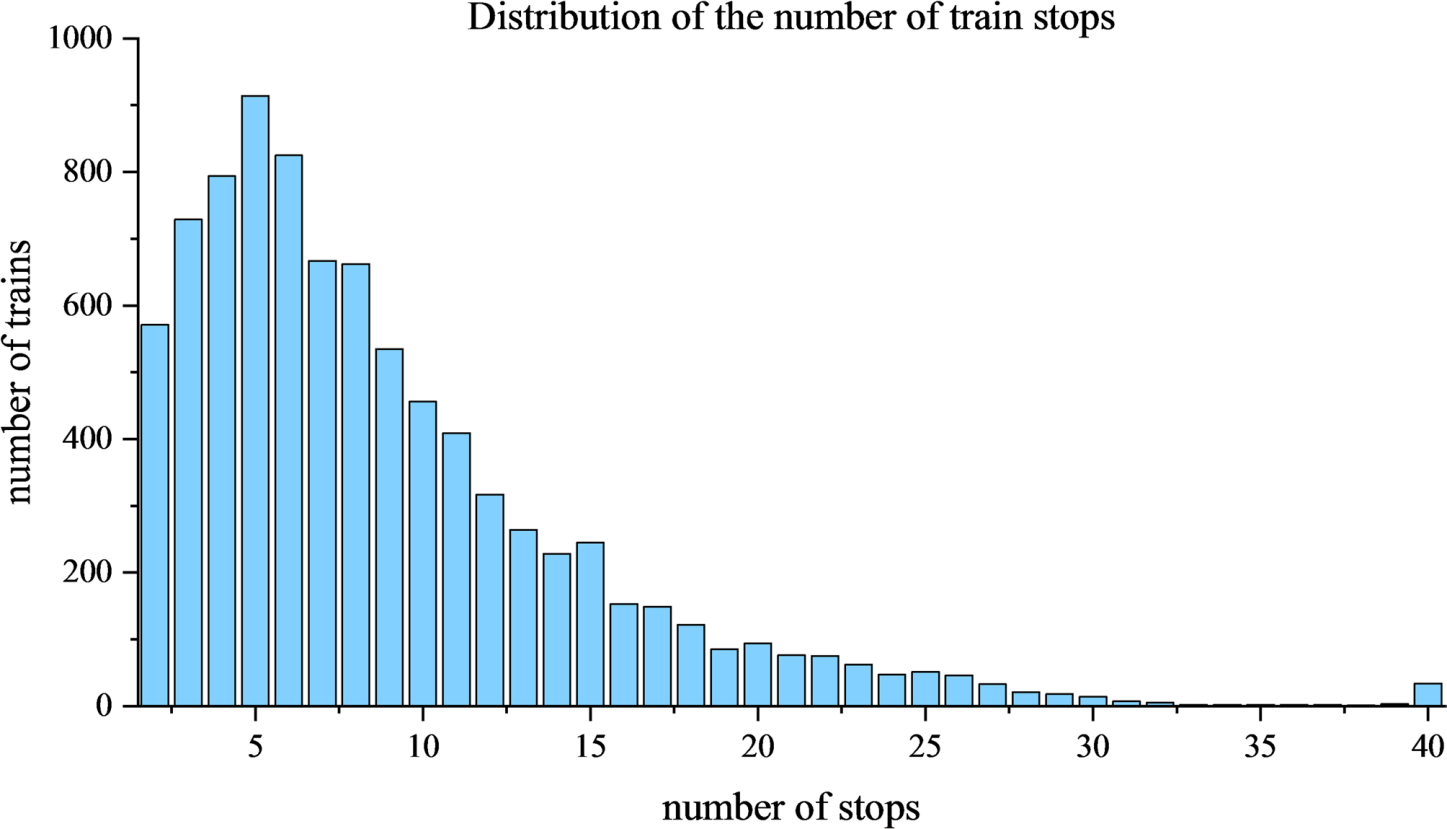
The distribution of the number of train stops.

In addition, as shown in Fig. 6, nearly 70% of trains stop at no more than 10 stations, while 914 trains stop at only 5 stations, indicating that trains with few stops dominate the organization of China’s railway system. The overall distribution exhibits a clear right-skewed characteristic: a large number of trains operate with short-stop sequences, while only a few trains have longer or more complex stop sequences. This distribution structure reflects the impact of passenger flow demand and trunk corridor organization on train timetables in my country; that is, the operating mode of most trains is mainly influenced by end-to-end (O-D) transportation demand, rather than by the demand for numerous intermediate stops. This observation also explains why the subsequent generation of passenger flows in this study is necessarily based on O–D demand as the primary modeling focus, since O–D movements constitute the dominant component of actual travel behavior in the national railway system.

### Demand network analysis

To construct the Demand Network, the Passenger Demand Estimation Method (PDEM) is first applied to simulate passenger demand across the entire HSR system. In real-world operations, China’s HSR services are predominantly operated with 8-car formations, supplemented by a smaller proportion of 16-car formations. Accordingly, this study assumes a nominal train capacity of 750 passengers, reflecting the approximate 3:1 ratio between 8-car and 16-car sets in practice. Based on this assumption, the PDEM simulation yields an estimated daily passenger volume of 8.77 million trips.

**Fig. 7:**
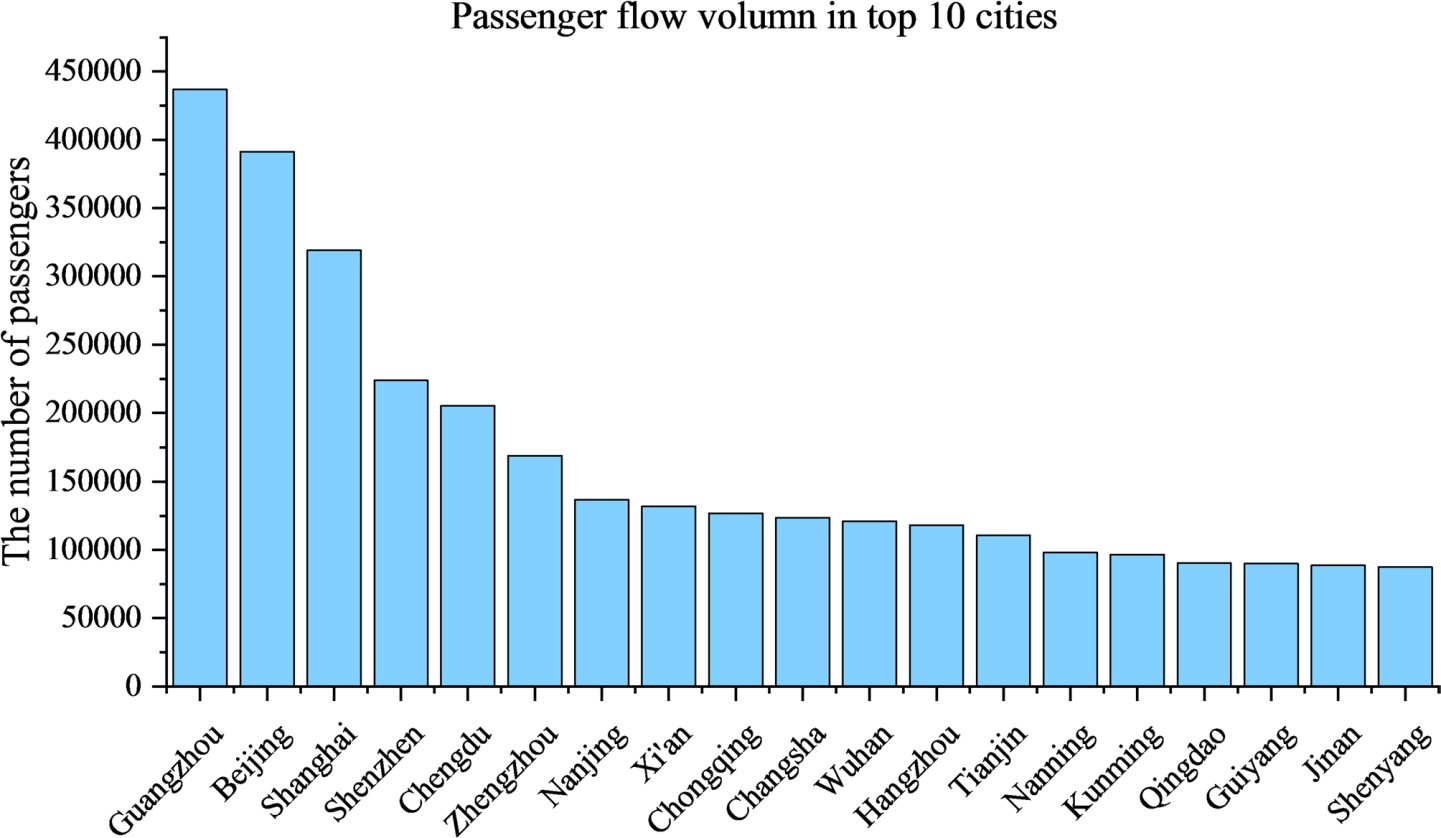
The passenger flow in top 20 cities.

For comparison, according to the *China Statistical Yearbook* (2025), the total number of EMU passengers nationwide in 2024 reached 3.272 billion, corresponding to an average of 8.94 million passengers per day. The difference between the PDEM-generated estimate and the official national statistics is therefore only 0.17 million passengers, representing an estimation error of merely 1.9%. Furthermore, as illustrated in Fig. 7, the simulated daily passenger volume at Guangzhou Station is approximately 0.44 million. According to the official statistics released by the Guangzhou Municipal Government on December 29, 2024 (https://www.gz.gov.cn/zwfw/zxfw/jtfw/content/post_10050315.html), the actual average daily passenger throughput of Guangzhou Station is around 0.47 million. This comparison demonstrates that the proposed method is not only accurate at the national scale but also capable of providing highly reliable passenger-demand estimations at the individual station level. At the same time, major hub cities such as Beijing, Guangzhou, Shanghai, Chengdu, Shenzhen, Zhengzhou, Nanjing, Xi’an, Chongqing, Changsha, Wuhan, Hangzhou, and Tianjin all handle more than 0.10 million passengers per day on average. The top 20 cities alone absorb approximately 36% of the total modeled passenger demand, despite representing less than 2% of all stations in the network. The vast majority of stations maintain passenger flow between 10,000 and 30,000.

**Fig. 8:**
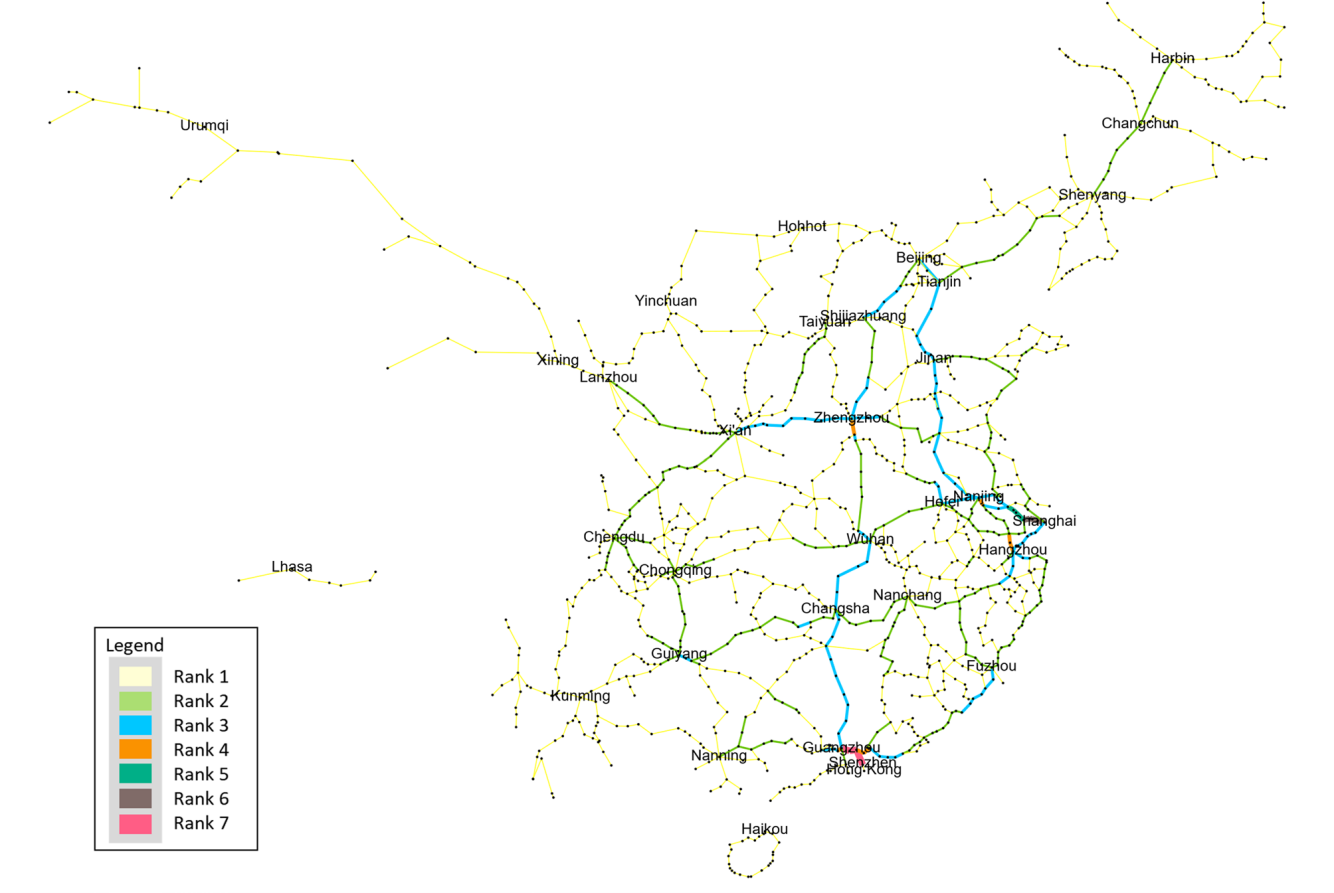
Demand network of HSR. The network visualization was generated using Gephi (version 0.9.2, https://gephi.org).

As shown in Fig. 8, beyond the aggregate validation, the spatial distribution of passenger flows in the Demand Network exhibits a distinctly heavy-tailed pattern at both the section and city levels. Section-level statistics show that the top 10% of railway corridors accommodate nearly 52% of the total simulated passenger volume, while the lowest 50% of sections collectively account for less than 12%. This concentration is visually reflected in the Rank-based flow map, where only a limited number of trunk corridors—primarily the Beijing–Guangzhou, Beijing–Shanghai, Yangtze River Delta, and Chengdu–Chongqing axes—appear in the top two ranks, indicating exceptionally high service pressure. In contrast, the majority of peripheral or regional connectors fall within the lowest three categories, underscoring their marginal contribution to nationwide OD mobility.

In summary, the concentration of passenger flow pressure in a few strategically located cities and sections reveals dependencies in the railway system that cannot be inferred from a purely physical perspective. From a vulnerability perspective, this means that disruptions to high-demand sections or major origin-destination hubs are likely to result in large-scale passenger volume losses across the entire railway system, while disruptions to low-demand sections will have only a relatively minor impact. Therefore, the demand network provides an indispensable quantitative basis for identifying critical service components and prioritizing vulnerability assessments in subsequent analyses.

### Sensitivity analysis

To evaluate the robustness of the proposed service-oriented vulnerability assessment framework, a one-factor-at-a-time sensitivity analysis was performed on three key parameters of the Passenger Demand Estimation Method (PDEM): the origin boarding proportion ${p_0}$, the minimum boarding volume ${V_{{\mathrm{min}}}}$, and the maximum boarding volume ${V_{{\mathrm{max}}}}$. Parameter robustness was assessed using (1) the relative sensitivity measure ${S_\theta }$, which quantifies the proportional change in the network-wide average vulnerability, and (2) Spearman’s rank correlation coefficient $\rho$, which evaluates the stability of the vulnerability ranking across all railway segments.

The results presented in Table 3 indicate that all parameters exhibit consistently low sensitivity, with ${S_\theta }$values ranging between 0.05 and 0.10, suggesting that moderate perturbations in PDEM inputs lead to only marginal changes in the overall vulnerability level. The boarding proportion ${p_0}$demonstrates the highest sensitivity (approximately 0.088–0.102), whereas ${V_{{\mathrm{min}}}}$and ${V_{{\mathrm{max}}}}$ show slightly lower responsiveness (approximately 0.05–0.07). This confirms that the macroscopic vulnerability metric is relatively insensitive to plausible variations in passenger distribution assumptions.

Moreover, Spearman’s correlation coefficients remain exceptionally high for all perturbations ($\rho >0.999$), confirming that the relative ranking of vulnerable railway segments is virtually unchanged under parameter variations. This stability indicates that the identification of critical and high-vulnerability links is highly robust with respect to uncertainties in passenger demand estimation.

Therefore, sensitivity analysis shows that the proposed framework can reliably assess service-oriented vulnerabilities. The results confirm that parameter uncertainties in the demand forecasting process have minimal impact on the degree and spatial distribution of high-speed rail network vulnerabilities.

**Table 3:** Sensitivity analysis.

Parameter	Value	${S_\theta }$	$\rho$
${p_0}$	0.75	0.101995	0.99991
${p_0}$	0.95	0.088276	0.999925
${V_{{\mathrm{max}}}}$	0.08	0.068207	0.99968
${V_{{\mathrm{max}}}}$	0.12	0.050899	0.999811
${V_{{\mathrm{min}}}}$	0.03	0.064784	0.999885
${V_{{\mathrm{min}}}}$	0.07	0.070833	0.999835

## Result and discussion

### Vulnerability of China’s HSR

With the construction of the physical network, functional network, and demand network, the proposed three-layer network model is fully established, enabling a comprehensive representation of both infrastructure connectivity and passenger flow dynamics. Based on the demand patterns captured in the third layer, this study further evaluates network vulnerability by examining the impacts on passenger flows when individual railway line segments become unavailable.

For each hypothetical disruption of a railway line segment, passenger transfer strategies are applied to estimate the resulting system-level passenger loss and the recoverable passenger volume through alternative services. These quantities provide a direct measure of the network’s ability to absorb operational shocks and to reallocate passenger demand via remaining connectivity. In this way, the vulnerability of each railway line segment is systematically quantified according to its impact on overall passenger flow performance.

**Fig. 9:**
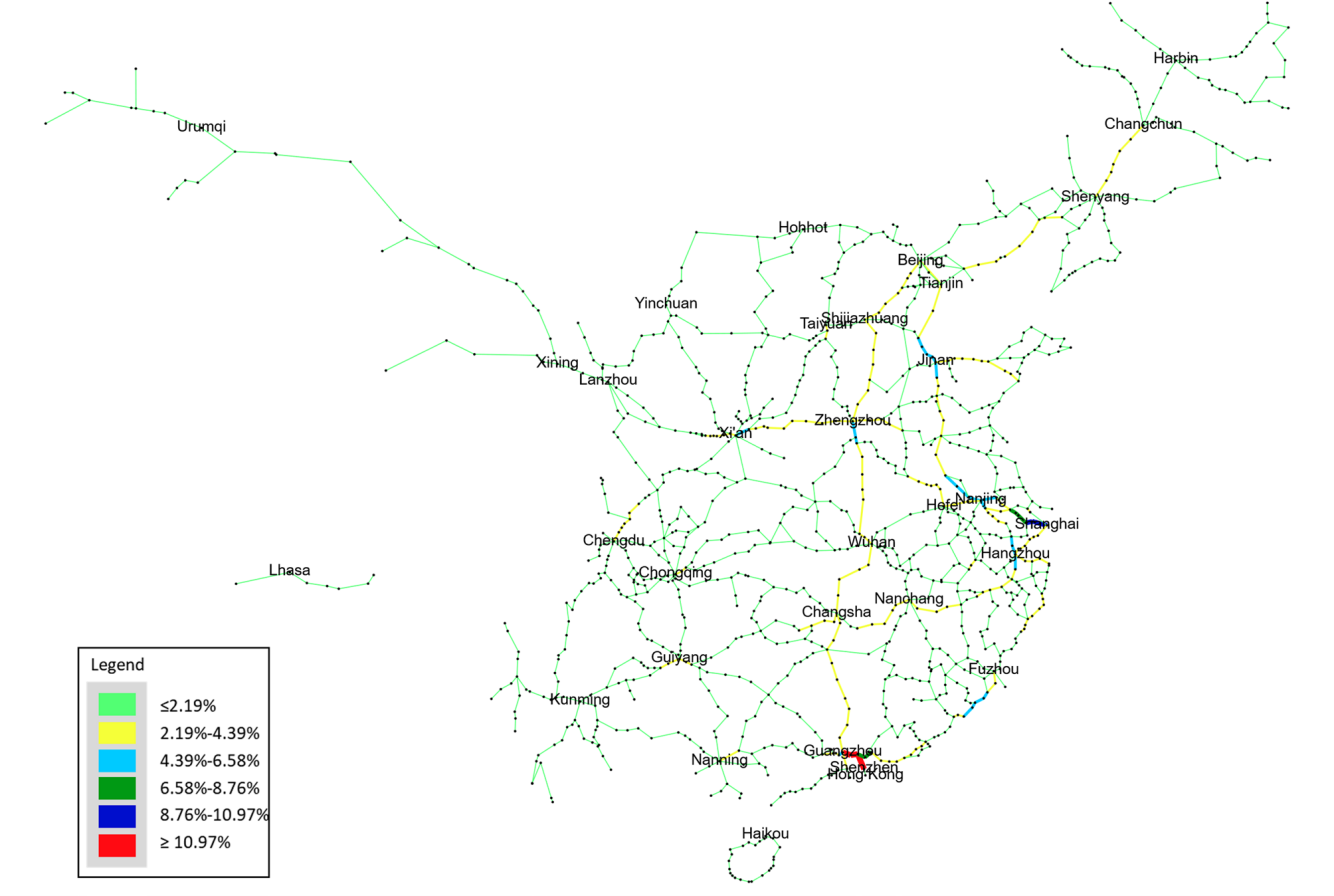
Vulnerability of each line in China’s high-speed railway network.

Figure 9 presents the spatial distribution of vulnerability across the entire network, highlighting specific corridors and line segments where service disruptions lead to a pronounced degradation of overall passenger transport capacity.

The network visualization was generated using Gephi (version 0.9.2, https://gephi.org).

As shown in Fig. 9, network segment vulnerability analysis reveals an extremely uneven distribution of systemic risks, with only a few corridors having a disproportionately large impact on the continuity of national passenger transport. Although the Guangzhou-Dongguan-Shenzhen route is relatively short, its high frequency and density of departures result in the highest service-based segment vulnerability. Following closely are segments along the Beijing-Zhengzhou-Wuhan-Guangzhou axis, the Shanghai-Hangzhou-Nanjing metropolitan area, and the Chengdu-Chongqing corridor, indicating that even with optimal transfer strategies, service disruptions on these routes will lead to significant passenger losses and significantly reduced recovery capabilities. The identification of these critical segments suggests that vulnerability stems not only from physical topology but also from the combined effects of concentrated passenger demand.

In contrast, most peripheral or regionally isolated sections are extremely vulnerable, reflecting both their limited role in nationwide originating transportation and their high substitutability within the surrounding network. Even sudden disruptions would not significantly impact the overall railway system. This contrast between core and peripheral areas highlights the hierarchical structure of China’s railway system, with long-distance passenger transport primarily relying on a few indispensable trunk lines.

Overall, the vulnerability patterns derived from the three-layer modeling framework provide important reference for strategic planning and operational risk management. Priority should be given to capacity enhancement, robust timetable design, and contingency response plans in high-vulnerability areas, as disruptions in these areas will have the most severe ripple effects on national transportation performance. These findings demonstrate the critical importance of integrating infrastructure, operational behavior, and demand dynamics when assessing railway system vulnerability, and highlight the value of the analytical methods employed in this study in ensuring the stability of large-scale transportation networks.

### Measures for mitigating HSR vulnerability

Network-level vulnerability patterns reveal the structural hierarchy and service dependencies of China’s high-speed rail system. However, a deeper understanding of vulnerability mitigation measures requires examining specific high-impact corridors under actual operational conditions. Particular attention should be paid to sections with high passenger volume, frequent service, and limited flexibility in route rerouting, as disruptions to these sections can disproportionately impact the entire network. To illustrate how the proposed framework can be applied to operational decisions and demonstrate the effectiveness of vulnerability reduction strategies in practice, this study further conducts a targeted analysis of a representative critical corridor within the network.

The Dezhou-Jinan section was chosen as a representative case because it is one of the service-critical sections identified in the national high-speed rail vulnerability assessment. Located on the northern section of the Beijing-Shanghai high-speed railway trunk line, this section experiences extremely high passenger volume, frequent train services, and limited available alternative routes. These characteristics make this section highly sensitive to emergencies: even short-term failures can cause severe operational losses, and recovery capabilities are limited. Therefore, this section provides a typical and representative scenario for demonstrating how the proposed mitigation measures can be applied to high-impact, high-demand sections within the national high-speed rail system.

Therefore, a disruption is assumed between Dezhou and Jinan. Based on the service-oriented vulnerability assessment, a comprehensive analysis of the daily operating schedules and transfer plans of the affected trains was conducted. The analysis results can provide specific recommendations for reducing line vulnerability from both topological and operational perspectives.

**Fig. 10:**
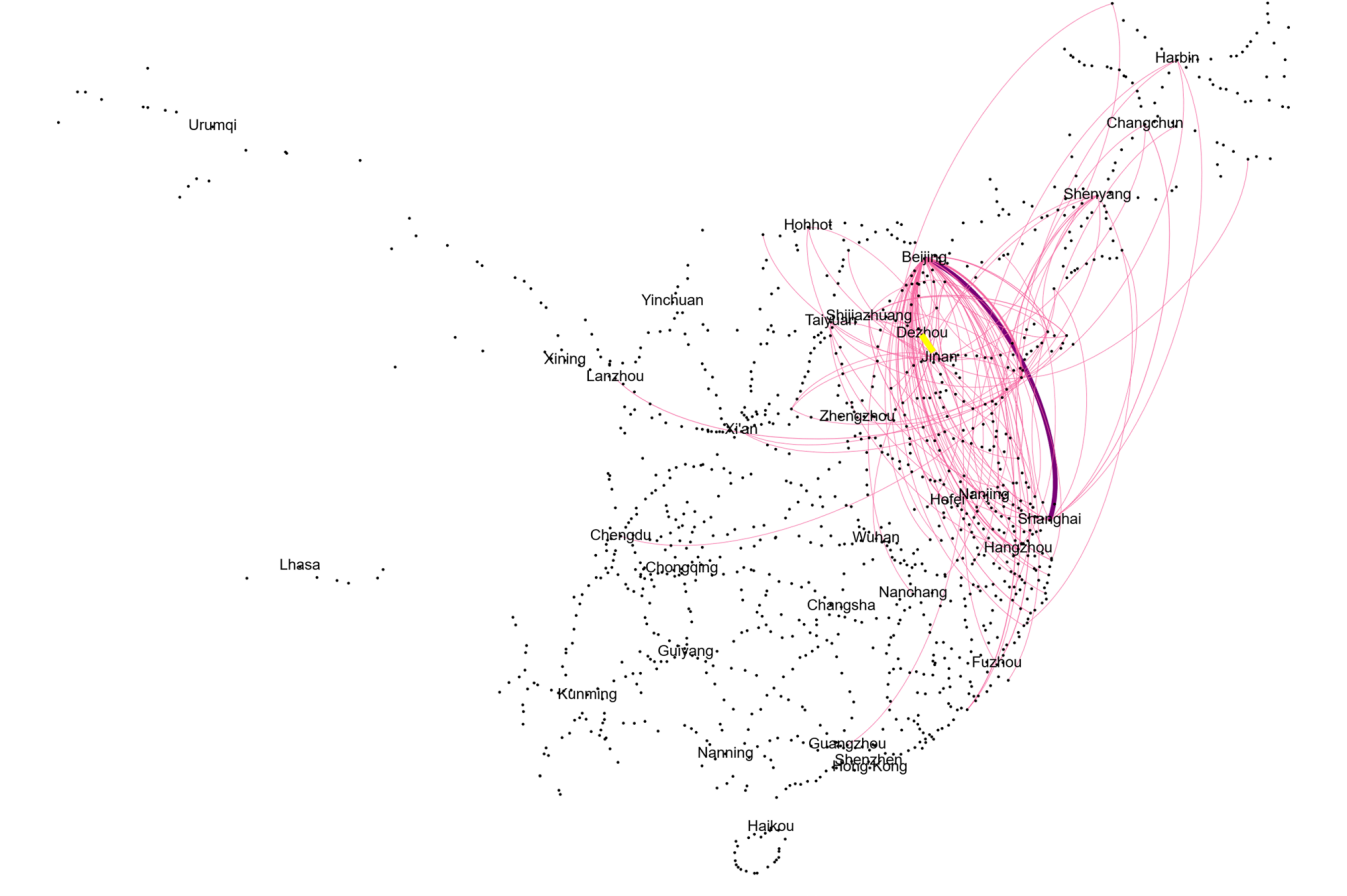
The OD distribution of affected trains online Dezhou-Jinan.

First, passenger traffic on the Dezhou-Jinan line, a crucial route connecting Beijing and Shanghai, was analyzed. This line has 385 trains operating, carrying approximately 510,000 passengers. Figure 10 shows the daily operating schedules of these trains; a wider curve indicates more trains connecting the two stations. Clearly, the Beijing-Shanghai route has the most trains, accounting for 22% of the total.

The network visualization was generated using Gephi (version 0.9.2, https://gephi.org).

When the line is attacked, 360 transfer plans are generated by the transfer algorithm, if 100 seats on each train are available for transferring, about 46,200 passengers could transfer by the other trains. Zhengzhou Station is the largest transfer station, with a total of 140 schemes transferring through Zhengzhou station.

**Fig. 11:**
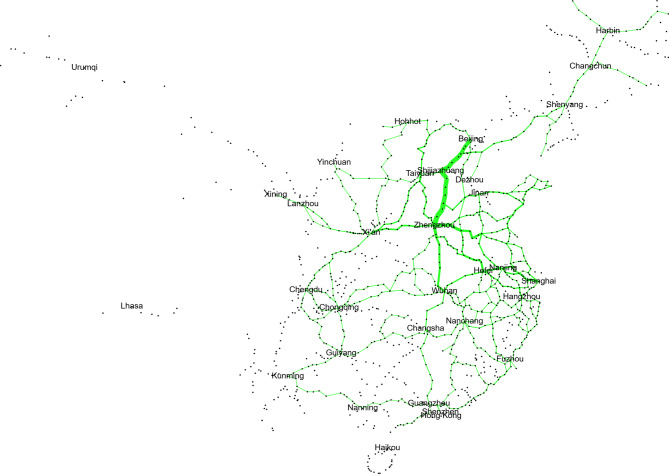
The path of trains for passengers to transfer. The network visualization was generated using Gephi (version 0.9.2, https://gephi.org).

Figure 11 shows the path of trains for passengers to transfer. The transfer path is shown as green, and the width indicates the quantity of transfer trains passing through the line. Most trains from Beijing to Zhengzhou are used for transfers. Combined with the OD distribution of the affected trains, it can be found that all trains departing from Beijing and its north need to transfer through the Beijing-Zhengzhou section. When the affected passengers arrive in Zhengzhou, there are several routes to reach their destinations.

From the perspective of topology, alternative railway is urgently needed to improve the vulnerability of line Dezhou-Jinan, an important line on section Beijing-Shanghai. As shown in Fig. 10, there are more than 300 trains from Dezhou to Jinan, an important line on section Beijing-Shanghai, the departure interval is less than 5 min. No other railway can carry such a large flow of passengers.

From the perspective of operation, the operator could adjust the path of the trains between Beijing and Shanghai within the railway capacity, the path Beijing-Zhengzhou-Wuhan-Shanghai and Beijing-Zhengzhou-Xuzhou-Nanjing-Shanghai are good alternative. Due to the limited railway capacity, this method is relatively ineffective in reducing vulnerability and is mainly used to provide services for passengers who are eager to travel.

In summary, the train disruption on the Dezhou-Jinan section exposed the inherent structural vulnerabilities and operational limitations of the current high-speed rail network. While transfer schemes and rerouting strategies can partially mitigate the impact by redistributing affected passengers to other lines, their effectiveness is limited by network capacity and heavy reliance on the Beijing-Shanghai corridor. Therefore, achieving long-term resilience requires combining infrastructure strengthening (such as constructing parallel sections or enhancing cross-regional connectivity) with dynamic operational strategies to flexibly adjust train routes in the event of disruptions. Integrating these measures will significantly enhance the system’s ability to maintain service continuity in the face of unexpected disturbances and reduce its vulnerability.

## Conclusion and suggestions

This paper firstly modelled the high-speed railway network as a three-layer network with the integration of physical network connection, transportation capacities and passenger flow. On this basis, a new service-oriented assessment method was proposed to evaluate the vulnerability of the HSR based on the travel service capacity for passengers before and after the uncertain interruptions. This method could assess the HSR vulnerability from the view of transportation service missions instead of using the pure complex network topological indicators in existing studies. In this model, a new passenger transfer strategy was proposed to calculate the remaining service capacities after the disruptions, and the transfer ratios of the affected passengers were estimated by a transfer algorithm. Finally, the case study of China’s HSR was conducted to evaluate the performance of the model, and the results can be summarized as following points.

1. The vulnerability of the lines in eastern China exhibits higher vulnerability than those in western regions as shown in the Fig. 9, while the density of railway network is precisely opposite. It indicates that the vulnerability is determined by the service capacity of the HSR, and the three-layer network proposed in this paper could reflect more realistically the ability of the HSR to resist the uncertain risk.
2. As the very important part of the Beijing-Shanghai line, the vulnerability of the Dezhou-Jinan line should be taken more attention. There are more than 300 trains pass through this line every day, and the alternate routes are all too far which will reduce the transfer intention of the passengers.
3. To effectively reduce the vulnerability of this busy section of the Beijing-Shanghai High-Speed ​​Railway, the construction of a second line should be considered. Analysis shows that the current Beijing-Shanghai High-Speed ​​Railway is under extremely high load and lacks sufficient alternative routes in the event of a failure. A parallel line would increase service redundancy and reduce the overall vulnerability of the high-speed rail system.

The results and conclusions may have some important implications for the construction of HSR and the adjustment of trains schedule to reduce the system vulnerability to deal with the sudden failure of railway lines.

1. The passengers are mapped to the vulnerable links based on the three-layer network, with which the manager could assess the distribution of passenger flow and prepare passenger evacuation plan in advance.
2. The interruption showed seasonal tendencies, especially for the meteorological disasters, the manager could adjust the trains schedule seasonally and ensure there are abundant redundancy of the trains in the transfer lines to absorb the passengers and reduce the system vulnerability.
3. The transfer cost should be considered in strategy design to reduce the system vulnerability. Taking the Beijing-Shanghai Line for example, the transfer routes are all too far. It is a good option to build a second line between Beijing and Shanghai to improve the redundancy of HSR according to the volume and distribution of passenger flow. Actually, the line is also being planned, and more similar lines could be planned and built between the emerging urban agglomerations.

However, there are some limitations in this study. First, it is assumed that the trains will stop the service when the line is interrupted, and the passengers transfer to other trains on other routes. In fact, the trains can be redistributed by the dispatch command system, which means parts of the passengers can be served by the original trains. Second, the transfer of passenger flow is not entirely in the HSR, and the supplementary role of aviation network, railway network and highway network on the transfer of passenger flow in the HSR is not considered in this paper. Finally, the interruption for the rail is simulated in this paper, and the actual attack such as the gale, rain and snow should be considered in the further study.

## Data Availability

The train timetable data used in this study were obtained from the Railway Customer Service Center of China (https://www.12306.cn). The railway infrastructure data were obtained from Baidu Map (https://api.map.baidu.com). National passenger statistics were collected from publicly available official sources, including the Statistical Bulletin on the Development of the Transportation Industry in 2024 published by the Ministry of Transport of the People’s Republic of China (https://xxgk.mot.gov.cn/2020/jigou/zhghs/202506/t20250610_4170228.html) and the China Statistical Yearbook 2024 published by the National Bureau of Statistics of China (https://www.stats.gov.cn/sj/ndsj/2024/indexch.htm).All data used in this study are publicly available from the respective official websites cited above.
